# Exosomal Serum Biomarkers as Predictors for Laryngeal Carcinoma †

**DOI:** 10.3390/cancers16112028

**Published:** 2024-05-27

**Authors:** Johannes Schuster, Olaf Wendler, Vanessa-Vivien Pesold, Michael Koch, Matti Sievert, Matthias Balk, Robin Rupp, Sarina Katrin Mueller

**Affiliations:** Department of Otolaryngology, Head and Neck Surgery, Friedrich-Alexander-Universität Erlangen-Nürnberg, Waldstrasse 1, 91054 Erlangen, Germany; johannes.schuster93@gmail.com (J.S.); olaf.wendler@uk-erlangen.de (O.W.); vpesold@gmail.com (V.-V.P.); michael.koch@uk-erlangen.de (M.K.); matti.sievert@uk-erlangen.de (M.S.); matthias.balk@uk-erlangen.de (M.B.); robin.rupp@uk-erlangen.de (R.R.)

**Keywords:** LSCC, exosomes, liquid-biopsy, biomarker, IGFBP7, ANXA1

## Abstract

**Simple Summary:**

Laryngeal squamous cell carcinomas (LSCC) are the third most common type of head and neck cancer. Approximately 40% of LSCC cases present with locally advanced carcinomas at diagnosis. The poor prognosis is mainly attributed to late diagnosis. Biomarkers capable of predicting LSCC at early stages are needed. We identified and validated exosomal insulin-like growth factor binding protein 7 (IGFBP7) and Annexin A1 (ANXA1) as promising exosomal biomarkers for LSCC detection.

**Abstract:**

Background: The lack of screening methods for LSCC is a critical issue, as treatment options and the treatment outcome greatly depend on the stage of LSCC at initial diagnosis. Therefore, the objective of this study was to identify potential exosomal serum biomarkers that can diagnose LSCC and distinguish between early- and late-stage disease. Methods: A multiplexed proteomic array was used to identify differentially expressed proteins in exosomes isolated from the serum samples of LSCC patients compared to the control group (septorhinoplasty, SRP). The most promising proteins for diagnosis and differentiation were calculated using biostatistical methods and were validated by immunohistochemistry (IHC), Western blots (WB), and ELISA. Results: Exosomal insulin-like growth factor binding protein 7 (IGFBP7) and Annexin A1 (ANXA1) were the most promising exosomal biomarkers for distinguishing between control and LSCC patients and also between different stages of LSCC (fold change up to 15.9, *p* < 0.001 for all). Conclusion: The identified proteins represent potentially novel non-invasive biomarkers. However, these results need to be validated in larger cohorts with a long-term follow-up. Exosomal biomarkers show a superior signal-to-noise ratio compared to whole serum and may therefore be an important tool for non-invasive biomarker profiling for laryngeal carcinoma in the future.

## 1. Introduction

LSCC is the third most common type of head and neck cancer [1]. All areas of the larynx (supraglottic, glottic, and subglottic) can be affected and lead to different symptoms. These symptoms and their onset, as well as treatment and prognosis, vary depending on the localization of the carcinoma [2,3]. Due to the nonspecific symptoms, especially of non-glottic laryngeal cancer, carcinomas are often not diagnosed until they are already at an advanced stage, resulting in delayed treatment [4]. Glottic carcinomas present relatively early causing voice changes and hoarseness. These symptoms occur even with small tumors. Hence, glottic carcinomas are usually diagnosed at an early stage. This results in a good prognosis due to the associated low probability of metastasis [2,5]. Supraglottic and subglottic carcinomas are usually diagnosed at advanced disease stages, as tumors localized in these subareas of the larynx tend to manifest symptoms with increasing local tumor extent. Consequently, these tumors present more often with nodal involvement at the time of diagnosis [2,6]. At the time of diagnosis, about 40% of supra- and subglottic lesions are already locally advanced carcinomas [6,7]. Overall, the proportion of patients, e.g., in Germany with T3 or T4 carcinoma and nodal involvement at initial diagnosis is 52–55% [8]. For the moment, chances of survival for patients with laryngeal cancer are closely related to the stage of disease at initial diagnosis. While early-stage T1 and T2 tumors show cure rates of up to 80% to 90%, the survival rate decreases to about 40% in patients with T3 and T4 carcinoma [9,10,11]. Preliminary and early stages of laryngeal carcinoma can be treated with minimally invasive surgery, providing very good treatment success and minimal functional restriction [12]. At present, surgical treatment of advanced carcinomas results in functional restriction or total loss of the organ (total laryngectomy) since surgeries preserving the structure and function of the larynx are rarely viable treatment options for advanced LSCC [9]. However, early diagnosis of LSCC is difficult since the laryngeal mucosa is not accessible for direct inspection and tissue changes cannot be directly detected either by the patient or the physician. Endoscopic or mirror examinations (followed by biopsy) must be performed in order to diagnose benign or malignant lesions. The use of serum as a liquid biopsy would provide an easily accessible option for diagnostic procedures that are accessible to almost any healthcare professional without the necessity of specialized training in laryngoscopy or other otolaryngology expertise. This could significantly simplify the detection of LSCC, allowing LSCC to be diagnosed more frequently at its early stages. This may result in better treatment options for patients, as the treatment outcome, survival rate, and quality of life after treatment are highly correlated with the stage at diagnosis. Therefore, non-invasive biomarkers that can diagnose LSCC and distinguish between early and late stages could significantly improve the diagnosis and treatment options and are urgently needed.

Several potential biomarkers for LSCC have been described, including long non-coding RNAs (lncRNAs), cell cycle regulators (Ki-67, cyclin D1, p27, p16, PCNA), apoptosis regulators (Bcl-2), tumor suppressor genes (p53), epidermal growth factor receptor (EGFR), and angiogenic, structural, and immunological markers (VEGF; E-cadherin, CD44; PD-L1). To date, however, there are no predictive biomarkers for the early detection or progression of LSCC [13]. Targeting EGFR has had an impact on LSCC and HNSCC therapy, but nevertheless, its usefulness for survival in advanced tumors remains limited [14]. No other biomarkers have influenced the management of HNSCC and LSCC [15]. Considerable efforts have been made to identify secreted biomarkers for different cancer types in serum or plasma, but with limited success. The enormous complexity of the samples and the large sample range make the detection of biomarkers in such body fluids considerably more difficult [16]. The potential biomarkers identified so far may lack clinical significance due to insufficient signal-to-noise ratios. The use of exosomes for detecting potential new biomarkers may offer an effective solution to this shortcoming.

Exosomes are small vesicles, measuring between 30 and 150 nm, that are secreted by virtually all cell types [17,18]. They contain a variety of proteins, lipids, DNA, mRNA, and microRNA that are representative of the cell from which they originate [19,20]. As exosomes represent the pathophysiologic state of their cell of origin, by containing information in the form of biomolecules, and being secreted into the bloodstream, they are a promising substrate for non-invasive liquid biopsy and early tumor detection [21]. Certain proteins associated with tumoral cell pathophysiology may be present in exosomes at an accumulated level. This accumulation of proteins may enable their detection as novel biomarkers [19,20,22]. Furthermore, malignant cells and cancer progression have been associated with increased exosome production and secretion [22]. Therefore, exosomes show promise as an analytical substrate to screen potential biomarkers for tumor detection and monitoring. However, isolation is time consuming and may not be available everywhere. We hypothesize that certain proteins associated with tumor development and progression might be present in exosomes in accumulated amounts and may be used for early diagnosis. Thus, the objectives of this study were (1) to identify novel non-invasive exosomal serum biomarkers for LSCC using a highly multiplexed approach; (2) to validate the most promising differentially expressed biomarkers in exosomes and whole serum; and (3) to analyze the potential of these biomarkers for the early detection of LSCC.

## 2. Methods and Materials

### 2.1. Patients

This study was a prospective observational study approved by the institutional review board of the Friedrich-Alexander-University of Erlangen-Nürnberg (No.: 20-451-B on 25 March 2021). All patients provided written informed consent prior to participating. Demographic data were collected prospectively from these patients.

Two different cohorts of LSCC were included in this study. The identification cohort (array analysis) consisted of *n* = 15 patients with an initial diagnosis of LSCC who had undergone panendoscopy for tumor staging and *n* = 7 control patients undergoing SRP. Corresponding clinical and pathologic data were retrieved for analysis. Patients receiving preoperative anticancer therapies and those with other concurrent or prior malignancies were excluded. Patients younger than 18 years were also excluded from this study. For cohorts A and B, the control group consisted of patients undergoing SRP for the treatment of an acquired nasal deformity. Patients who had undergone surgery to correct functional pathologies such as the treatment of nasal obstruction, OSAS, or inflammatory changes were excluded, as were SRP revisions, in order to provide a control group of healthy individuals. Patients in the same age group as the tumor patients were selected for the control group. The validation cohort (WB, ELISA) consisted of a total of *n* = 75 LSCC and *n* = 54 control patients (SRP). The samples of cohort A were also included in cohort B.

### 2.2. Three-Step Study Design

The study design included a three-step process to identify biomarkers and to validate them in a separate patient cohort. First, a multiplexed proteomic array (*n* = 2000 proteins) on exosomes isolated from serum was performed on the identification cohort. Second, ELISAs of exosomes isolated from serum and ELISAs of whole serum samples were conducted for IGFBP7 and ANXA1. These validations were performed in exosomes and serum in order to optimize the signal-to-noise ratio (exosomes) and point-of-care use (whole serum). Third, IGFBP7 and ANXA1 were analyzed in tissue. IHC was performed on paraffin-embedded tissue sections from the patients whose serum samples were used for array analysis to confirm the presence and localization of the proteins of interest in tumoral tissue. Additionally, WBs were run on tissue lysates from a subgroup of the validation cohort, consisting of LSCC (*n* = 8) and control (SRP; *n* = 6) patients.

#### 2.2.1. Serum Collection

Serum samples were taken the day before surgery. After the collection of whole blood, the tubes were kept at room temperature for 30 min, followed by centrifugation at 3000× *g* for 10 min. The resulting supernatant was collected and aliquoted to be stored at −80 °C until further examination.

#### 2.2.2. Serum Pool Groups

Before exosome isolation, the serum samples of cohort A were categorized into five serum pool groups: four samples from patients with T1N0 LSCC, two from patients with T2N0 LSCC, three from patients with T4 (N0) LSCC, and six from patients with T4 (N+) LSCC. The control group consisted of seven samples from patients undergoing SRP. Each pool consisted of 300 µL serum. The respective patients were selected to ensure that there were no significant differences in terms of age, gender, or comorbidities. In addition, pooling matched patients allows for equalizing individual differences.

#### 2.2.3. Exosome Purification from Serum

Exosomes were isolated from serum samples of the validation cohort. The exosome purification procedure was performed using an Exo-spin blood kit, following the protocol for serum samples (Protocol Version 7.0). The Exo-spin technology combines two standard methods: precipitation followed by size-exclusion chromatography (SEC). Purification via SEC can separate large portions of non-exosomal proteins, resulting in a clean exosome fraction (Rider et al., 2016; Zhang et al., 2020) [23,24].

Three hundred (300) µL of each serum pool was used for purification. After centrifugation at 300× *g* for 10 min, the supernatant was centrifuged at 16,000× *g* for 30 min. The supernatant was then diluted 2:1 with Exo-spin buffer. The diluted sample was incubated at room temperature for 15 min and mixed by inversion of the tube. Centrifugation was performed at 10,000× *g* for 60 min. The supernatant was discarded, and the remaining pellet was resuspended with 100 µL PBS (Exo-spin kit). Exo-spin size-exclusion chromatography (SEC) columns were equilibrated at room temperature for 15 min prior to use. The preservation buffer was removed, and each column was reconstituted with 250 µL of PBS. One hundred (100) µL of resuspended exosomes were applied to each Exo-spin column. The flow-through was discarded. Two hundred (200) µL of PBS pH8 was added to elute the exosomes. The columns were then centrifugated at 50× *g* for 60 s. Exosome lysates were prepared by adding NP40, EDTA, and NaCl to a final concentration of 0.5% NP40, 1 mM EDTA, and 250 mM NaCl. The total protein content was quantified using a micro bicinchoninic acid (BCA) protein assay kit (Thermo Fisher Scientific, Bonn, Germany). For the proteomic array, 400 µg of protein was used per pool sample.

#### 2.2.4. Multiplexed Proteomic Array of Serum Pool Exosomes

Proteomic analysis was performed using the RayBio L-Series Human Antibody Array 2000 Glass Slide Kit (AAH-BLG-2000-8, Raybiotech, Peachtree Corners, GA, USA) according to the manufacturer’s recommended protocol. Purified exosome samples containing 400 µg of total protein were used in the incubation step. The data analysis by fluorescence was provided by Raybiotech. Median normalization of the data was performed using the analysis tool provided by Raybiotech. Known exosome markers that were also analyzed with the antibody array were used as references for further normalization, via geNorm. These exosome markers were Annexin V, MRP 1/CD9, HSP70, EpCAM, and ICAM-1/CD54. Background signals were reduced by subtracting the lowest negative value per subarray from all other values of the same subarray. The fold change (FC) of the mean values and median values were calculated.

The proteins selected for further validation were chosen based on FC, physiological function, and previous association with LSCC and/or HNC in the literature. A table of the complete array analysis is included in the supplementary data (Supplementary Data S1).

#### 2.2.5. Validation of Selected Proteomic Results by Enzyme-Linked-Immunosorbent-Assay (ELISA)

ELISAs were performed on exosomes and whole serum for IGFBP7 and ANXA1. For whole serum analysis, *n* = 75 LSCC and *n* = 54 controls (cohort B) were tested individually. ELISAs were performed according to the manufacturer’s protocols. For IGFBP7, the serum was diluted 1:2–1:4 and exosome lysates were diluted 1:4 with Reagent Diluent. The Human IGFBP7 DuoSet ELISA No. DY1334 (R&D Systems, Minneapolis, MN, USA) was used. For Annexin A1, the serum was diluted 1:2 and exosome lysates were diluted 1:2 with Sample Diluent. The Human Annexin A1 PicoKine ELISA No. EK1745 (Boster Biological Technology, Pleasanton, CA, USA) was used. Standard curves were generated, and results were calculated by normalizing the values on total protein concentration of the tissue and exosome samples (BCA assay, Thermo Fisher Scientific, Bonn, Germany).

#### 2.2.6. Analysis of IGFBP7 and ANXA1 in Tissue

##### Tissue Analysis by Immunohistochemistry (IHC)

To localize IGFBP7 and ANXA1 in LSCC tissue, IHC was performed on tissue sections from cohort A. IHC on paraffin-embedded sections was performed using ZytoChem-Plus AP Polymer Kit (Zytomed Systems GmbH, Berlin, Germany). To make the epitopes available for antibody binding, the sections underwent deparaffinization and heat-mediated antigen retrieval by either phosphate citrate buffer or Tris-EDTA buffer at 95 °C for 20 min. The additional reduction of background staining was achieved by BLOXALL™ Endogenous Peroxidase and Alkaline Phosphatase Blocking Solution (Vector Laboratories, Inc., Newark, NJ, USA) for 10 min before the protein block with blocking solution (included in the kit). The antibodies listed in Table 1 were incubated overnight at 4 °C. A nonspecific antibody (Cell Signaling Technology, Inc., Danvers, MA, USA) served as a negative control. Afterwards, the ZytoChem-Plus AP reagent was applied. Antigens were stained with SIGMAFAST™ Fast Red TR/Naphthol AS-MX tablets. Counterstaining was performed with Harris’ hematoxylin solution (ORSAtec GmbH, Bobingen, Germany). The sections were covered with Aquatex (Merck, Darmstadt, Germany). Tissue sections were photographed using a BZ-X810 microscope with BZ-X800 viewer and analyzer software (Keyence, Germany, Neu-Isenburg). Images were taken at 20× and 40× magnification. Scale bars were included. Contrast and brightness were adjusted.

##### Tissue Protein Extraction

Tissue proteins were extracted from samples using T-PER Tissue Protein Extraction Reagent (Thermo Fisher Scientific, Bonn, Germany). Thirty (30) mg of tumor or control tissue was transferred to a 500 µL solution of TPER and protease inhibitor cocktail (Carl Roth, Karlsruhe, Germany). Homogenization of the samples was achieved using Ultra Turrax Homogenizer (IKA^®^-Werke GmbH & CO. KG, Staufen, Germany). The resulting samples were rotated at 4 °C for 2 h during incubation. The supernatant was collected after centrifugation at 10,000× *g* for 30 min at 4 °C, and the total protein amount was quantified using a bicinchoninic acid (BCA) protein assay kit (Thermo Fisher Scientific, Bonn, Germany).

##### Validation via Western Blots (WB)

To validate the results of the array analysis, WBs were performed on tissue lysates from 14 patients of the validation cohort. The tumor samples consisted of T1, T2, T3, and T4 tumors (*n* = 2 each). A total of *n* = 8 LSCC and *n* = 6 control tissue lysate samples were analyzed. GAPDH was used as reference protein for normalization. For each lane, 30 µg of tissue lysate samples were used. After denaturation at 95 °C for 5 min with loading buffer, samples were run on gradient 8% to 15% SDS-PAGE and transferred to nitrocellulose membranes (ROTI^®^NC; Carl Roth, Karlsruhe, Germany). The primary detection antibodies listed in Table 1 were incubated overnight, followed by the secondary antibodies, peroxidase-labeled anti-mouse/rabbit immunoglobulin G (IgG) antibody (Thermo Fisher Scientific, Bonn, Germany). The blots were incubated with SuperSignal West Dura Extended Duration Substrate or (Thermo Fisher Scientific, Bonn, Germany) and the signals were imaged using ChemStudio PLUS (Analytik Jena, Jena, Germany). Band intensity was quantified using VisionWorks version 8.2 (Analytik Jena, Jena, Germany). Results were normalized to GAPDH.

### 2.3. Statistical Analysis

For the descriptive statistics, the analysis of the biomarker levels, and the creation of graphics, GraphPad Prism Version 9.3.1 (GraphPad Software, La Jolla, CA, USA) was used. To distinguish LSCC patients from healthy subjects, receiver operating characteristic curves for IGFBP7 and ANXA1 were calculated using GraphPad Prism Version 9.3.1 (GraphPad Software, La Jolla, CA, USA). For the analysis of the measured biomarker levels, all data were first checked for a normal distribution using the Shapiro-Wilk test. All data were nonparametric. Differences between the groups were determined using the Kruskal-Wallis-test. *p*-values < 0.05 were considered as statistically significant.

## 3. Results

### 3.1. Demographics

Clinicopathological data for all patients enrolled in the study are presented in Table 2. A total of *n* = 15 patients with LSCC and *n* = 7 controls (SRP) were included in the identification cohort (cohort A). Tumors of different sizes (T1–T4) and nodal involvement (N0-N3b) were included. A total of *n* = 75 patients with LSCC and *n* = 54 controls (SRP) were included in the validation cohort (cohort B). The samples of cohort A were also included in cohort B.

In cohort A there were no significant differences between the LSCC and the control group (LSCC *n* = 15, and control *n* = 7) regarding gender, race, comorbidities, or medication.

Furthermore, there was no significant difference between LSCC patients of cohorts A and B with regard to any of the comorbidities mentioned. This also applies to the comparison of control patients from cohorts A and B, with the exception of cardiovascular disease.

In cohort B, the age of LSCC patients was significantly higher compared to control patients (*p* < 0.001). Significantly more men than women were included in the LSCC group in comparison to the control group (*p* < 0.001). Comorbidities like metabolic diseases, cardiac disease, nicotine and alcohol consumption, and COPD occurred significantly more often in the tumor group.

For cohorts A and B, the control groups consisted of SRP patients. This explains the significant age gap and differences in the male to female ratio between LSCC and the control group in cohort B. This age gap is considered to be the main reason for differences in comorbidities and medication. The differences in the male to female ratio are common in LSCC. Therefore, statistically significant differences between tumor patients and healthy controls are to be expected.

### 3.2. Identification of IGFBP7 and Annexin A1 as Promising Differentially Expressed Exosomal Proteins Using a Highly Multiplexed Proteomic Array Analysis

In the first step, a proteomic multiplexed array analysis was used to identify promising novel exosomal biomarkers (Table 3). The exosomal proteins IGFBP7 and ANXA1 were selected based on the most promising fold change and physiological function. Both proteins were overexpressed at all tumor stages compared to the control group. The highest fold change for both proteins was found in T1 and T2 LSCC/control (IGFBP7 FC: T1 = 3.48, T2 = 5.93; ANXA1 FC: T1 = 10.25, T2 = 15.91).

Proteomic results of IGFBP7 and AXNA1 are shown in Table 3 (see Supplementary Data S1 for results of all proteins of the proteomic analysis). Out of 2000 proteins examined on the antibody array, we chose IGFBP7 and ANXA1 out of the most differentially expressed proteins for further validation. The proteins (of the array analysis) selected for further validation as potential biomarkers were chosen based on FC, physiological function, and previous association with LSCC and/or HNC in the literature.

### 3.3. Identification and Validation Cohort Show Matching Results for IGFBP7 and Annexin A1

The identification cohort showed the overexpression of IGFBP7 and ANXA1 regarding all tumor stages versus controls, in exosomes isolated from serum. The highest tumor-to-control ratio (fold change) was found in T1 and T2 carcinomas for both IGFBP7 and ANXA1 in the array analysis.

ELISAs were run on exosomes isolated from serum and whole serum samples to measure IGFBP7 and ANXA1 in the validation cohort. ELISAs were performed in exosomes and serum in order to optimize the signal-to-noise ratio (exosomes) and point-of-care use (whole serum).

**Figure 1:**
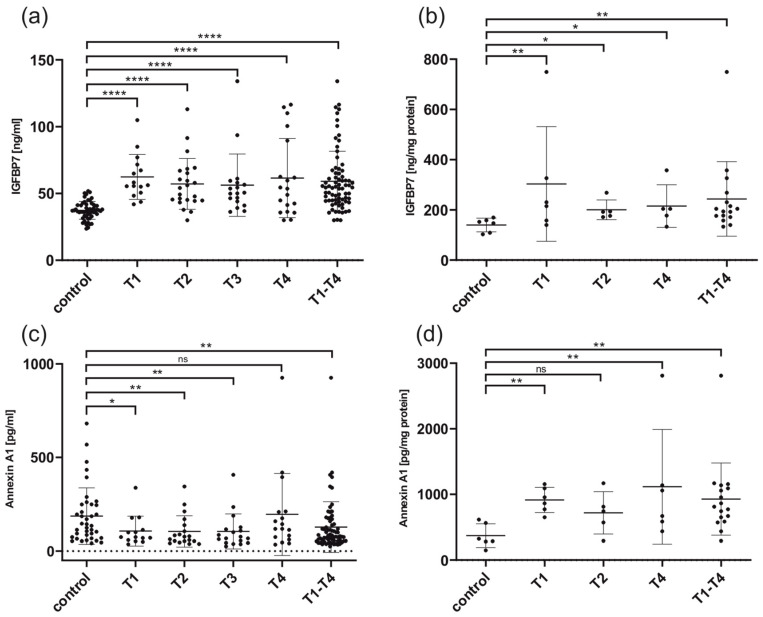
ELISA results for IGFBP7 and ANXA1 on whole serum and exosomes. (**a**) IGFBP7 on whole serum comparing control patients and different stages of LSCC. For all tumor sizes, IGFBP7 showed a significant overexpression in LSCC compared to controls. (**b**) IGFBP7 on exosomes isolated from serum comparing control patients to different stages of LSCC. IGFPB7 showed significant overexpression for all tumor sizes compared to controls. (**c**) ANXA1 on whole serum comparing control patients and different stages of LSCC. For all tumor sizes, except T4, ANXA1 showed a significant downregulation in LSCC compared to controls. (**d**) ANXA1 on exosomes isolated from serum comparing control patients to different stages of LSCC. For all tumor sizes, except T2, ANXA showed significant overexpression compared to controls. * *p* < 0.05; ** *p* < 0.01; **** *p* < 0.0001; ns, not significant. For the calculation of ELISA results and the creation of graphics, GraphPad Prism Version 9.3.1 (GraphPad Software, La Jolla, CA, USA) was used. Differences between the groups were determined using the Kruskal-Wallis-test. *p*-values < 0.05 were considered as statistically significant.

IGFBP7 was significantly increased in the whole serum of LSCC patients compared to controls (T1–T4: FC = 1.6; *p* < 0.0001). IGFBP7 serum levels of every T stage (T1–T4) were significantly overexpressed in comparison to the serum of healthy individuals (T1: FC = 1.7, *p* < 0.0001; T2: FC = 1.5, *p* < 0.0001; T3: FC = 1.5, *p* < 0.0001; T4: FC = 1.7, *p* < 0.0001; Figure 1). This was also true for exosomes (T1–T4: FC = 1.7, *p* < 0.01; Figure 1).

Annexin A1 was significantly downregulated in the whole serum of all tumor sizes compared to controls, except T4 (T1: FC = 0.6, *p* < 0.05; T2: FC = 0.6, *p* < 0.01; T3: FC = 0.6, *p* < 0.01; T4: FC = 1.0, *p* = ns; Figure 1) ELISAs on exosomes, on the other hand, did show overexpression of ANXA1 in tumor samples compared to controls. The overexpression in exosomes was significant for all tumor sizes except T2. The overexpression of ANXA1 in exosomes measured via ELISA matches our results in the array analysis of exosomes (T1: FC = 2.5, *p* < 0.01; T2: FC = 1.9, *p* = n.s.; T4: FC = 3.0, *p* < 0.01; T1–T4: FC = 2.5, *p* < 0.01; Figure 1).

**Figure 2:**
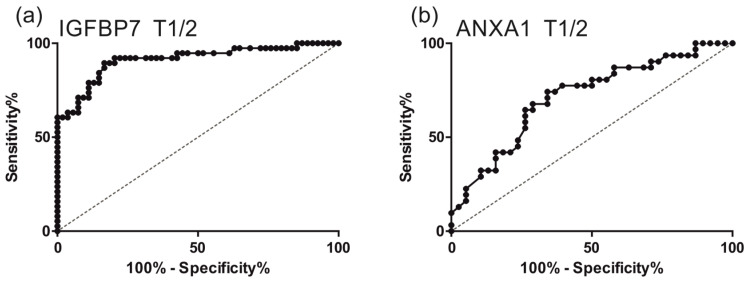
Receiver operating characteristic (ROC) curve to distinguish LSCC (T1 and T2) patients from healthy subjects by IGFBP7 (**a**) and ANXA1 (**b**). (**a**) ROC curve for IGFBP7: area under the ROC curve (AUC) 0.9128; standard error a 0.03235; 95% confidence interval b 0.8494 to 0.9762; significance level *p* < 0.0001. (**b**) ROC curve for ANXA1: area under the ROC curve (AUC) 0.7148; 0standard error a 0.0623; 95% confidence interval b 0.5927 to 0.8369; significance level *p* = 0.0023. ROC curves for IGFBP7 and ANXA1 were calculated using GraphPad Prism Version 9.3.1 (GraphPad Software, La Jolla, CA, USA).

As we were screening for biomarkers that are applicable for the early detection of LSCC, early tumor stages were of particular interest (T1, T2). We calculated the specificity and sensitivity of the potential biomarkers IGFBP7 and ANXA1 via ROC analysis for T1 and T2 tumors using the ELISA analysis data (Figure 2). For IGFBP7 with the cut-off value of 41.85, sensitivity was 92.1% and specificity was 79.6%. For ANXA1 with the cut-off value of 99.5, sensitivity was 71.0% and specificity was 65.8%.

The cut-off values were selected to achieve a high sensitivity rate. A high sensitivity rate and a low false-negative rate are particularly important when screening for malignant diseases, as false-negative results must be minimized. In cancer screening, it is particularly important that patients are not incorrectly classified as cancer free.

### 3.4. Analysis of IGFBP7 and ANXA1 in Tissue

#### 3.4.1. Immunohistochemical Patterns for IGFBP7 and Annexin A1

IHC for IGFP7 and ANXA1 was performed on the tissue samples of LSCC patients from cohort A. Control IHC was run on malignancy-free larynx tissue.

**Figure 3:**
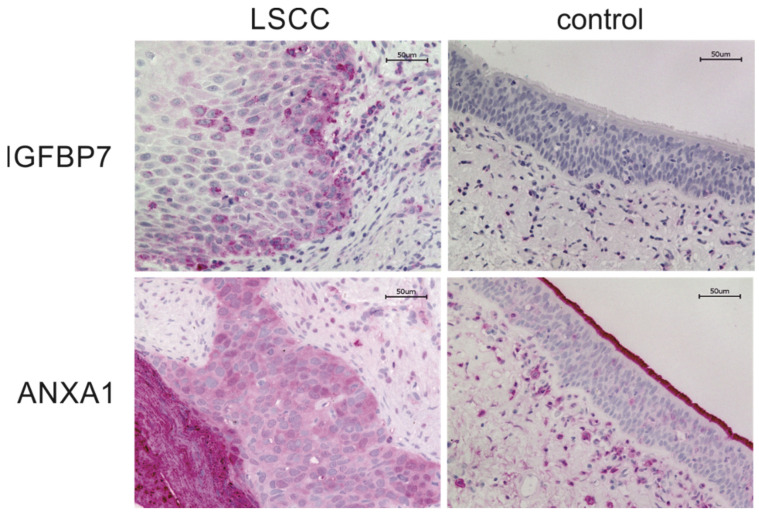
Immunohistochemistry of LSCC and control tissue for IGFBP7 and ANXA1. Tissue sections were photographed using a BZ-X810 microscope with BZ-X800 viewer and analyzer software (Keyence, Germany, Neu-Isenburg). Images were taken at 20× and 40× magnification. Scale bars were included. Contrast and brightness were adjusted.

IGFBP7 was mainly located at the invasion front of the tumor. In the stroma surrounding the tumor tissue, weak staining could be observed for some inflammatory cells and fibroblasts. In normal tissue, epithelium showed no staining for IGFBP7. Some inflammatory cells and fibroblasts were stained (Figure 3).

Annexin A1 showed strong staining of the tumor tissue, especially in the keratinized part of the carcinoma. The surrounding tissue was lightly stained; some inflammatory cells and fibroblasts also showed stronger staining. Normal tissue showed light staining. Inflammatory cells and fibroblasts showed stronger staining and stood out compared to stroma and epithelium. The brush border and mucus layer of the epithelium showed the strongest staining (Figure 3).

LSCC showed strong IGFBP7 expression primarily in the invasion front of the tumor. The staining was weaker in the midst of the tumor. Control tissue without malignancy showed a weak staining in some inflammatory cells and fibroblasts and no staining in the epithelium.

LSCC showed strong ANXA1 expression in the whole tumor tissue, especially in the keratinized part of the carcinoma. The surrounding tissue was slightly stained; some inflammatory cells and fibroblasts also showed stronger staining. Control tissue showed light staining. The epithelium and the underlying connective tissue stroma were stained slightly. Inflammatory cells and fibroblasts showed stronger staining and stood out compared to stroma and epithelium. The brush border and mucus layer of the epithelium showed the strongest staining.

#### 3.4.2. Validation of Proteomic Results Using Western Blots

Western blots were performed on tissue lysates from 14 patients of the validation cohort. The tumor samples consisted of T1, T2, T3, and T4 tumors (*n* = 2 each). In total *n* = 8 LSCC and *n* = 6 control tissue lysate samples (malignancy-free tumor-distant tissue) were analyzed for IGFBP7 and ANXA1.

**Figure 4:**
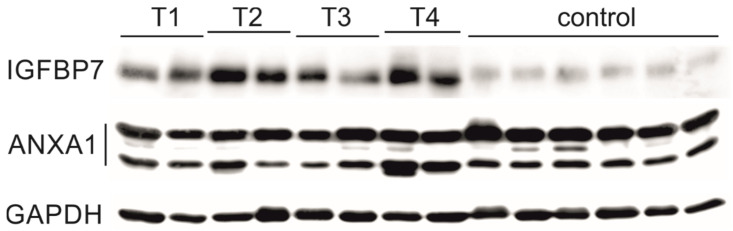
Western blots on tissue lysates of different stages of LSCC (T1–T4) and controls for IGFBP7 and ANXA1. In total *n* = 8 LSCC and *n* = 6 control tissue lysate samples were analyzed for IGFBP7 and ANXA1. WBs signals were imaged using ChemStudio PLUS (Analytik Jena, Jena, Germany). Band intensity was quantified using VisionWorks version 8.2 (Analytik Jena, Jena, Germany). Results were normalized to GAPDH. Semiquantitative densitometric analysis of the band patterns and normalization to GAPDH yielded the FC value of 3.81 for IGFBP7 and 0.84 for Annexin A1. IGFBP7: All stages of LSCC showed a stronger staining in comparison to controls. ANXA1: Western blots on tissue lysates of different stages of LSCC and controls for ANXA1. The band pattern of ANAX1 showed hardly any differences between the tumor and the control samples. GAPDH: Reference protein for WB analysis showing a consistent band intensity for all samples.

**Figure 5:**
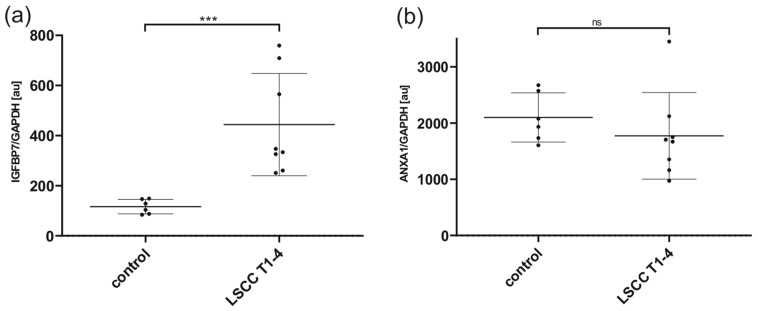
Quantitative, densitometric analysis of Western blot results are presented in Figure 4. The absolute signal intensities of the respective samples were normalized to the absolute signal intensities of GAPDH. In total *n* = 8 LSCC and *n* = 6 control tissue lysate samples were analyzed for IGFBP7 and ANXA1. Signals were imaged using ChemStudio PLUS (Analytik Jena, Jena, Germany). Densitometric analysis was performed by band intensity quantification via the image processing software VisionWorks version 8.2 (Analytik Jena, Jena, Germany). Results were normalized to GAPDH. Abbreviations: au, arbitrary units. *** *p* < 0.001; ns, not significant.

The band pattern of IGFBP7 showed an increased band intensity in all examined tumor lysates, independent of stage, compared to the control samples. This finding indicates that IGFBP7 is overexpressed in the tumor tissue and matches the proteomic array analysis, IHC and ELISA results (Figure 4). The band pattern of Annexin A1 showed hardly any differences between the tumor and the control tissue (Figure 4). Semiquantitative densitometric analysis of the band patterns and normalization to GAPDH yielded the FC value of 3.81 for IGFBP7 and 0.84 for Annexin A1(Figure 5).

## 4. Discussion

LSCC is the third most common type of HNSCC [1]. Despite an increase in the overall survival rate of LSCC patients, the prognosis for those with advanced laryngeal cancer has not improved in recent decades [25]. This is due to the nonspecific symptoms of laryngeal cancer, which often lead to late diagnosis and delayed treatment [4]. Currently, there are no non-invasive biomarkers available for the early diagnosis of LSCC. Therefore, we analyzed exosomal serum biomarkers for LSCC to address this shortcoming. Ideally, a combination of biomarkers should be used to decrease interindividual differences [26].

Exosomes are a promising substrate for noninvasive liquid biopsy and early tumor detection as they represent the pathophysiological state of their cell of origin. Increased exosome production has been associated with malignant cells and cancer progression. Certain proteins associated with tumoral cell pathophysiology can accumulate in exosomes, enabling their detection as novel biomarkers [19,20,22]. Additionally, exosomal biomarkers exhibit a superior signal-to-noise-ratio compared to whole serum. Therefore, our group decided to use exosomal proteins as the substrate for the identification cohort to screen potential novel biomarkers. The objective of our group was to identify biomarkers that can be tested or monitored via serum analysis, as exosome isolation is time-consuming and may not be implemented into clinical practice.

IGFBP7 and ANXA1 were identified as potential novel exosomal biomarkers for LSCC by multiplexed array analysis. Our results showed increased levels of IGFBP7 and ANXA1 in LSCC compared to controls for all tumor stages. The FC for IGFBP7 of T1 (3.48) and T2 (5.93) and for ANXA1 of T1 (10.25) and T2 (15.91) are especially noteworthy.

IGFBP7 is a member of the IGFBP family, involved in the regulation of cell proliferation, angiogenesis, apoptosis, and mesenchymal transition [27,28,29,30,31]. It has been reported in multiple carcinomas, showing variable expression depending on the tumor site. For example, it acts as cancer promoting in esophageal carcinoma [32], gastric cancer [33], and head and neck squamous cell carcinomas (HNSCC) [15,34]. Downregulated expression of IGFBP7 was reported in liver carcinomas [28,35], lung cancer [36,37,38] and prostate cancer [39,40].

ANXA1 functions as a calcium-dependent phospholipid-binding protein with anti-inflammatory properties. It acts as a glucocorticoid-regulated protein, inhibiting phospholipase A2 (PLA2) during inflammatory responses, thereby reducing the release of arachidonic acid [41,42]. ANXA1 is expressed in multiple tissues and is involved in diverse cellular processes, including cell proliferation, differentiation, survival, apoptosis, migration, and angiogenesis. The deregulation of ANXA1 is frequently associated with cancer development, contributing to tumor initiation, proliferation, and metastasis [43]. ANXA1 expression has been reported to correlate with the development of hepatocellular carcinoma (HCC) [44], colorectal cancer (CRC) [45,46], lung cancer [47], pancreatic cancer [48], melanoma [49] and skin cancer [50]. ANXA1 expression has been reported to be negatively correlated with prostate cancer, esophageal squamous cell carcinoma (ESCC) [51,52], oral squamous cell carcinoma [53,54], cervical cancer [55], laryngeal cancer [56,57], nasopharyngeal cancer [58], and breast cancer [59].

### 4.1. Tissue Results for IGFBP7 and ANXA1

Our results in tissue demonstrated increased expression of IGFBP7 and ANXA1 in LSCC compared to controls, as confirmed by both IHC and WB. Both proteins showed strong staining in the tumoral tissue, while the surrounding normal tissue displayed light or no staining. IGFBP7 was predominantly expressed in the invasion front of the tumor. Both IGFBP7 and ANXA1 displayed light staining in the inflammatory cells and fibroblasts. The WB band pattern of IGFBP7 showed an increased band intensity in all examined tumor lysates compared to the control samples, independent of tumor stage. This finding suggests that IGFBP7 is overexpressed in the tumor tissue and is consistent with the proteomic array analysis, IHC, and ELISA results.

The band pattern of ANXA1 showed hardly any differences between the tumor and the control tissue. A slight reduction in band intensity was detectable for tumors. Semiquantitative densitometric analysis of the band patterns and normalization to GAPDH yielded the FC value of 3.81 for IGFBP7 and 0.84 for ANXA1. The IHC of ANXA1 showed that physiologic epithelium, connective tissue stroma, fibroblasts, and inflammatory cells express ANXA1 to a certain extent. The lack of differences in expression between tumor and control samples for ANXA1 may be due to tissue lysates being a mixture of different cells. Due to the heterogeneity of tumor tissue, the proportion of different cell types in the tumor lysates and therefore the extent of ANXA1 expression may vary. Furthermore, the control tissues utilized for Western blot analysis were obtained from areas distant from the tumor. This means that there may still be a meaningful presence of inflammatory cells, which could result in higher levels of ANXA1 in the controls.

The literature regarding ANXA1 in LSCC is limited. While some studies reported increased ANXA1 levels in LSCC [60,61], others showed a decrease in ANXA1 expression in LSCC [56,57,62,63]. In the broader context of HNSCC, the majority of studies suggest a reduction in ANXA1 expression [61,62,64,65,66]. Some studies directly link the reduction or loss of ANXA1 in HSCC to tumor development and other HNSCC-related oncogenes such as EGFR [62,64] and miR-196a/b [65,66]. Consistent with our IHC results, Deng et al. found high expression of Annexin A1, A2 and A4 in human laryngeal carcinoma tissues using IHC, despite these annexins not being detected by differential proteomic analysis. The group suggested that low abundance proteins like annexins may not be easily detectable during proteomics analysis due to limited sensitivity [60]. In contrast, a study by Alves et al. using serial analysis of gene expression (SAGE) revealed a significant decrease in ANXA1 expression in LSCC. IHC confirmed the reduced expression of ANXA1 in the nucleus and cytoplasm of larynx tumors compared to normal tissue, while an increase in ANXA1 expression was detected in the membrane of tumorous versus normal tissue [57]. Other studies reported a downregulation of ANXA1 expression in laryngeal tumor tissue, with a concurrent increase in ANXA1 in inflammatory cells situated within the tumor microenvironment [56,63].

ANXA1 has been reported in inflammatory cells, particularly in mast cells that are involved in inflammatory responses [56,63,67,68]. Our IHC results also showed ANXA1 expression in inflammatory cells. The increase in degranulated mast cells observed in laryngeal tumors and peritumoral tissue indicates that these tumor cells recruit and activate mast cells to release biological mediators that may be harmful to the tumor or contribute to tumor development. In response to chemoattractant substances released by tumor cells, inflammatory cells, including mast cells, accumulate at sites of tumor growth. Depending on local stromal conditions, they could either promote or inhibit tumor growth by altering the peritumoral micromovement [63].

### 4.2. ELISA Results for IGFBP7 Matching Previous Findings in LSCC

Our ELISA results showed a significant overexpression of IGFBP7 in the serum and exosomes of LSCC patients compared to controls, matching our results in tissue. IGFBP7 was significantly overexpressed in the serum of LSCC patients compared to controls (T1–T4: FC = 1.6; *p* < 0.0001). This was also true for exosomes (T1–T4: FC = 1.7, *p* < 0.01; Figure 1).

Sepiashvili et al. investigated hypopharynx (FaDu) and larynx (UTSCC8, UTSCC42a) cancer cell lines by mass spectrometry-based proteomic profiling and gene expression microarrays. This was followed by the verification of selected markers through quantitative real-time PCR, WBs, IHC, and ELISAs [15]. The study demonstrated significantly increased expression of IGFBP7 in LSCC by IHC and WBs. Furthermore, significantly increased levels of IGFBP7 were detected in the plasma of LSCC patients compared to healthy individuals via ELISA [15]. Our tissue results also showed overexpression of IGFBP7 in WBs and IHC, and significantly increased levels of IGFBP7 in the serum of LSCC for all tumors (T1–T4: FC = 1.6; *p* < 0.0001). In conjunction with these results, our findings in exosomes and whole serum underline the potential of IGFBP7 as a noninvasive biomarker. Moreover, IGFBP7 may be able to differentiate not only between individuals with and without cancer but also between early and late disease stages.

### 4.3. Potential Influence of Comorbidities on IGFBP7 Expression

In patients affected by ischemic heart disease (IHD), Lisowska et al. found an average 1.25-fold increase in IGFBP7 serum levels in comparison to the population (1.76 ± 1 ng/mL vs. 1.43 ± 0.44 ng/mL, respectively, *p* = 0.019) [69]. Regarding our study, most of the patients grouped under the category of cardiovascular diseases exhibited solely arterial hypertension. Only a subset of these patients had coronary artery disease, myocardial infarction, or had undergone stenting or bypass surgery, which would suggest a diagnosis of IHD. Three of 15 LSCC patients in cohorts A and eight of 53 LSCC patients in cohort B could therefore be classified as having IHD. None of the control patients of cohort A and B had been diagnosed with IHD. The ELISA results of our study showed a 1.7-fold increase in IGFBP7 serum levels in comparison to the control group for all tumor stages. The p value was >0.0001. This means that in our ELISA results, the serum level difference of IGFP7 in LSCC patients compared to the control group was approximately 45% higher than the serum IGFBP7 level difference of IHD patients compared to the population group in the study by Lisowska et al. Due to the small number of patients with IHD in the tumor group of cohorts A and B and the significantly lower fold change in IGFBP7 levels in IHD patients compared to our results in LSCC patients, it can be assumed that the influence of this comorbidity on our ELISA results is probably minimal at worst.

Ruan et al. showed that serum IGFBP7 levels are elevated during acute exacerbations of COPD. After clinical recovery, IGFBP7 levels decreased [70]. Only seven out of 53 LSCC patients and zero control patients in cohort B had COPD. None of these patients had an acute exacerbation of COPD at the time of blood sampling.

### 4.4. ELISA Results of ANXA1 Show Opposing Data for Exosomes and Serum

Annexin A1 was significantly downregulated in the serum of all tumor stages compared to controls, except T4 (T1: FC = 0.6, *p* < 0.05; T2: FC = 0.6, *p* < 0.01; T3: FC = 0.6, *p* < 0.01; T4: FC = 1.0, *p* = ns; Figure 1). ELISAs on exosomes, on the other hand, did show significant overexpression of ANXA1 in tumor samples compared to controls except T2 (T1: FC = 2.5, *p* < 0.01; T2: FC = 1.9, *p* = n.s.; T4: FC = 3.0, *p* < 0.01; T1–T4: FC = 2.5, *p* < 0.01; Figure 1).

To the best of our knowledge, there is no literature regarding ANXA1 serum levels in LSCC. Our study is the first to investigate ANXA1 in the blood of LSCC patients compared to healthy controls. This is why the only possible comparison is with results from other tumor types. In a study of lung cancer patients, Rong et al. detected that serum Annexin A1 levels were significantly higher than in patients with benign lung diseases and healthy control patients via ELISA analysis. The group also observed that lung cancer with a high serum level of Annexin A1 is more likely to show an aggressive phenotype, i.e., poorly differentiated to undifferentiated lung cancer and lymphatic metastasis. These results suggest the possibility that serum ANXA1 may be a risk factor for lung cancer and a potential biomarker [71]. Han et al. found that serum ANXA1 in ESCC patients was significantly lower than in control patients (*p* < 0.001) but increased after chemoradiotherapy (*p* < 0.001) [52]. This matches our findings of decreased serum levels of ANXA1 in LSCC patients.

### 4.5. Differences in ANXA1 Expression between Exosomes and Serum

Our data on ANXA1 showed opposing results in exosomes and whole serum. It is known from other entities that results in exosomes and whole serum may be opposed due to the different secretion mechanism of exosomes [72]. Furthermore, there is conflicting literature about the expression levels of ANXA1 in tissue as described above [56,57,60,61,62,63,64,65,66]. ANXA1 has been described as a “double-sided” protein due to its diverse and sometimes opposing functions. The function of ANXA1 may be specific to each tumor type due to post-translational modifications of the protein impacting expression across a range of cell and cancer types. Furthermore, its influence on cancer may depend on its differential distribution among the cytoplasm, nucleus, and cell surface [73]. Several groups have described the occurrence of three subcellular localizations of ANXA1 as being nuclear, cytoplasmic, and associated with the plasma membrane. Depending on the specific site, ANXA1 may participate in tumorigenesis in different ways [42,74,75]. An increased presence of ANXA1 in cell nuclei is considered a significant predictor of poor overall survival in oral and esophageal squamous cell carcinoma [76,77]. In esophageal cancer, ANXA1 expression was found to be decreased in the cytosol and membrane but overexpressed in the nuclei. This suggests that the subcellular localization of ANXA1 may play an important role in tumorigenesis, in addition to its overall expression level [78]. The membrane association of ANXA1 enables the interaction with formyl-peptide-receptors (FPRs). Via the stimulation of FPRs, ANXA1 can induce oncogenic pathways. This has been reported in cases of gastric cancer [79] and breast cancer [80]. MDX-124, a humanized anti-ANXA1 monoclonal antibody, has been shown to suppress cell proliferation significantly in ANXA1-expressing breast, ovarian, pancreatic, colon, and lung cancer cell lines (*p* < 0.013). MDX-124 disrupted the interaction of ANXA1 with FPR1/2. Furthermore, the reduced cell proliferation induced by the antibody was mediated by arresting the cell cycle progression in the G1 phase instead of inducing apoptosis. In addition, MDX-124 significantly inhibited tumor growth in both TNBC (4T1-LUC triple-negative breast cancer) and pancreatic cancer syngeneic mouse models (*p* < 0.0001) [81]. ANXA1 overexpression in tumor cell cytoplasm has been reported in various cancers, including breast cancer and squamous cell carcinoma [82]. ANXA1 may also be seen as a promising biomarker for the diagnosis of LSCC. However, due to its contradictory activity in cancer in general and in LSCC, further research is needed in order to determine the clinical meaningfulness of ANXA1.

### 4.6. Limitations

The study was based at a single center in Germany, which may limit the generalizability of the findings to the wider population. Furthermore, due to the small sample size, continued experimental studies involving a larger number of samples of LSCC patients and molecular mechanisms are required to reach a definitive conclusion. In cohort A, there are no significant differences between the LSCC and the control group regarding gender, race, and comorbidities. At the same time, the control group of cohort B differs significantly from the tumor group in terms of age, gender, and comorbidity. This limits the comparability of these two groups. Therefore, the preliminary results need to be confirmed by a prospective study including a larger number of subjects as well as by the functional analysis of IGFBP7 and ANXA1 through in vitro studies. A detailed understanding of the function and importance of IGFBP7 and ANXA1 may help to further elucidate the biological mechanisms of laryngeal carcinoma and support the development of early diagnosis and preventive treatment.

It is important to note that tumor markers can be altered by certain comorbidities. There are studies that show an association between specific comorbidities and IGFBP7 or ANXA1 [69,70,83]. As blood samples for our study were taken from all patients the day before surgery or panendoscopy as part of the standard preoperative examination, acute inflammatory processes or exacerbations of disease can be reliably excluded, as the patients would otherwise not have been applicable for the operation.

## 5. Conclusions

IGFBP7 and ANXA1 were identified as potential novel non-invasive protein biomarkers for the early detection of LSCC by the combination of a highly multiplexed proteomic approach, WB, IHC, and ELISAs. IGFBP7 and ANXA1 may be potential predictors for LSCC, especially for early disease stages. Further studies as well as larger numbers of LSCC subjects are needed to investigate the clinical meaningfulness.

## Figures and Tables

**Table 1:** Primary antibodies used for WBs and IHC.

Method	Target	Host	Class	Immunogen	Clone	Manufacturer
IHC, western blot	IGFBP7	mouse	monoclonal	IGFBP7 aa 181–282	H-3	Santa Cruz, Heidelberg, Germany
IHC	IGFBP7	rabbit	polyclonal	peptide	-	Proteintech, Planegg, Germany
IHC, western blot	Annexin A1	rabbit	polyclonal	recombinant protein aa 1–346	-	Proteintech, Planegg, Germany
IHC	Annexin A1	mouse	monoclonal	recombinant protein aa 1–346	1E1B7	Proteintech, Planegg, Germany
Western blot	GAPDH	mouse	monoclonal	recombinant protein aa 1–335	1E6D9	Proteintech, Planegg, Germany

Abbreviations: IHC, immunohistochemistry; WB, Western blot; aa, amino acids.

**Table 2:** Demographics for LSCC and control patients. Cohort A: *n* = 15 LSCC and *n* = 7 controls (SRP) for proteome analysis; Cohort B: *n* = 75 LSCC and *n* = 54 controls (SRP) for validation by ELISA and WBs; in some cases, not all data were available. Mean values and standard deviation were calculated using Microsoft Excel (Version 2404, Redmond, WA, USA), *p*-values were determined using GraphPad Prism (Version 9.3.1, La Jolla, CA, USA).

Characteristics in (%)	LSCC	Control	*p*
Cohort A	15	7	-
Mean age in years (±SD)	63.4 (±9.8)	55.4 (±7.1)	ns (*p* = 0.069)
Gender			
Male	13/15 (86.7)	5/7 (71.4)	ns (*p* = 0.388)
Female	2/15 (13.3)	2/7 (28.6)	ns (*p* = 0.388)
Tumor size (T)			
T1	4/15 (26.7)	-	-
T2	2/15 (13.3)	-	-
T3	0/15	-	-
T4	9/15 (60.0)	-	-
Nodal status (N)			
N0	9/15 (60.0)	-	-
N1	0/15	-	-
N2	3/15 (20.0)	-	-
N3	3/15 (20.0)	-	-
Distant metastasis (M)			
M0	15/15 (100)	-	-
M+	0/15	-	-
Caucasian	15/15 (100)	7/7 (100)	-
Comorbidity			
Metabolic disease	5/15 (33.3)	0/7	ns (*p* = 0.082)
Cardiovascular disease	9/15 (60.0)	3/7 (42.9)	ns (*p* = 0.452)
Smoker	10/15 (66.7)	2/7 (28.6)	ns (*p* = 0.095)
Alcohol	12/15 (80.0)	4/7 (57.1)	ns (*p* = 0.262)
COPD	3/15 (20.0)	0/7	ns (*p* = 0.203)
OSAS	1/15 (6.7)	0/7	ns (*p* = 0.484)
Medication			
Antihypertensive medication	8/15 (53.3)	3/7 (42.9)	ns (*p* = 0.647)
Antidepressants	2/15 (13.3)	0/7	ns (*p* = 0.311)
Opioids	0/15	0/7	-
Immunosuppressants	0/15	0/7	-
Cohort B	75	54	-
Mean age in years (± SD)	61.4 (± 9.2)	34.5 (± 13.8)	*p* < 0.001
Gender			
Male	66/75 (88.0)	32/54 (59.3)	*p* < 0.001
Female	9/75 (12.0)	22/54 (40.7)	*p* < 0.001
Tumor size (T)			
T1	15/75 (20.0)	-	-
T2	23/75 (30.7)	-	-
T3	18/75 (24.0)	-	-
T4	19/75 (25.3)	-	-
Nodal status (N)			
N0	37/75 (49.3)	-	-
N1	7/75 (9.3)	-	-
N2	17/75 (22.7)	-	-
N3	5/75 (6.7)	-	-
Distant metastasis (M)			
M0	75	-	-
Race			
Caucasian	75/75 (100)	53/54 (98.1)	
Comorbidity			
Metabolic disease	19/53 (35.8)	3/54 (5.6)	*p* < 0.001
Cardiovascular disease	23/53 (43.4)	1/54 (1.9)	*p* < 0.001
Smoker	46/59 (78.0)	20/54 (37.0)	*p* < 0.001
Alcohol	43/59 (72.9)	28/54 (51.9)	*p* = 0.021
COPD	7/53 (13.2)	0/54	*p* = 0.006
OSAS	1/53 (1.9)	0/54	ns (*p* = 0.311)
Medication			
Antihypertensive medication	21/53 (39.6)	2/54 (3.7)	*p* < 0.001
Antidepressants	3/53 (5.7)	1/54 (1.9)	ns (*p* = 0.299)
Opioids	0/53	1/54 (1.9)	ns (*p* = 0.32)
Immunosuppressants	1/53 (1.9)	0/54	ns (*p* = 0.311)

Abbreviations: LSCC, laryngeal squamous cell carcinoma; art. HTN, arterial hypertension; COPD, chronic obstructive pulmonary disease; OSAS, obstructive sleep apnea syndrome; SD, standard deviation; ns, not significant.

**Table 3:** Multiplexed array results showing the fold changes between LSCC and control patients. The FC of IGFBP7 and ANXA1 for different stages of LSCC are displayed (see Supplementary Data S1 for results of all proteins of the proteomic analysis). Tumor samples included *n* = 4 patients with T1N0 LSCC, *n* = 2 patients with T2N0 LSCC, *n* = 3 patients with T4 (N0) LSCC, and *n* = 6 patients with T4 (N+) LSCC. The control group consisted of *n* = 7 samples from patients undergoing SRP. FCs were calculated by dividing the mean values of the tumor groups by the mean value of the control group. Data analysis was provided by Raybiotech. Median normalization of the data was performed using the analysis tool provided by Raybiotech. Known exosome markers that were also analyzed with the antibody array were used as references for further normalization, via geNorm.

Protein	T1 (N0)/C	T2 (N0)/C	T4 (N0)/C	T4 (N+)/C
IGFBP7	3.48	5.93	1.52	1.17
Annexin A1	10.25	15.91	1.58	4.21

Abbreviations: IGFBP7, Insulin-like growth factor binding protein 7.

## Data Availability

The data that support the findings of this study are available from the corresponding author, upon reasonable request.

## Supplementary Materials

The following supporting information can be downloaded at: https://www.mdpi.com/article/10.3390/cancers16112028/s1.
