# Supplementary material for: Exosomal Serum Biomarkers as Predictors for Laryngeal Carcinoma
Source: Cancers (Basel). 2024 May 27;16(11):2028. doi: 10.3390/cancers16112028 (PMC11171163; doi:10.3390/cancers16112028)
Supplement: Supplementary file 1 [file cancers-16-02028-s001.zip › cancers-2969668-supplementary.pdf]

Protein	FC (MV LSCC/MV control				
	T1(N0)/C	T2(N0)/C	T4(N0)/C	T4(N2)/C	T4(N3b)/C
TNF RI / TNFRSF1A	27,21	60,94	1,79	8,08	4,37
Thrombospondin-2	1,26	3,07	1,58	8,05	3,48
DR3 / TNFRSF25	18,51	33,59	1,85	7,58	0,00
MYL3	10,38	3,14	0,69	7,05	0,41
Tie-1	13,98	16,95	2,50	5,59	0,51
uPAR	12,39	10,02	1,86	4,40	0,89
NOV / CCN3	14,46	22,85	1,58	4,37	0,82
Plakophilin 1	1,60	4,91	1,93	4,37	2,83
ERRa	1,41	1,24	1,20	4,23	1,52
TRAIL R4 / TNFRSF10D	18,74	28,89	2,32	3,98	1,62
TRADD	2,88	5,20	1,44	3,84	2,86
ROBO4	1,87	1,35	2,93	3,74	1,91
Annexin A1	10,25	15,91	1,58	3,66	4,74
TGF-beta 3	7,66	9,14	1,75	3,64	0,36
Thymopoietin	13,09	7,44	0,74	3,61	1,16
Netrin-4	1,14	1,57	1,67	3,49	1,54
SSEA-4	0,49	0,48	0,95	3,45	0,83
TRPS1	9,89	6,89	1,42	3,45	0,71
Presenilin 1	1,25	4,16	1,37	3,44	0,84
Der p2	2,23	3,87	0,48	3,43	0,62
Serpin A12	0,73	5,33	1,81	3,34	5,63
VEGF R2 (KDR)	13,26	16,03	1,26	3,34	1,06
PEPSINOGEN I	8,79	19,89	1,07	3,25	0,85
DMP-1	6,77	10,46	1,78	3,23	1,07
CDA	15,83	2,48	7,66	3,22	14,57
TREM-1	7,04	9,09	1,12	3,20	0,33
CARHSP1	4,08	0,41	1,43	3,20	4,63
HSPA8	0,33	1,91	0,45	3,16	0,72
MMP-14	7,12	7,22	1,40	3,15	0,67
XEDAR	10,67	8,86	1,40	3,15	1,23
Tie-2	10,85	10,26	1,10	3,15	0,99
ACPP	8,25	9,56	1,59	3,12	0,89
GRP75	0,76	0,96	0,89	3,07	0,77
TGF-beta RII	15,93	20,59	0,78	3,06	0,92
COMP	2,61	2,59	3,31	3,01	4,75
Plexin B1	7,96	6,85	1,24	3,00	0,89
uPA	1,23	1,78	1,54	2,98	1,53
VEGF R1	5,77	10,50	1,32	2,95	0,72
Kallikrein 11	0,35	0,81	0,76	2,95	0,83
HSP40	1,04	0,61	1,76	2,94	1,13
AKR1C3	9,25	13,10	1,18	2,90	1,98
IFN-alpha / beta R1	6,72	9,93	1,17	2,80	0,32
IL-12 p70	6,51	10,50	0,75	2,75	0,72
APJ	0,58	0,60	0,43	2,75	0,71
ME1	5,01	4,62	0,54	2,69	1,01

**Leptin (OB)**	9,84	10,34	0,84	2,68	0,92
**TACE**	6,53	12,29	1,66	2,68	0,64
**Krt73**	5,27	1,14	0,44	2,64	0,81
**Gastrokine 1**	7,04	8,21	1,64	2,64	1,62
**TRAP220**	6,01	6,57	1,10	2,64	0,93
**RPL17**	8,44	8,46	0,95	2,59	0,76
**MYH6**	6,71	15,21	0,47	2,59	20,24
**Chromogranin B**	7,81	11,99	1,13	2,58	2,85
**ANGPTL8**	6,66	7,56	1,54	2,58	3,75
**GSTM1**	5,30	3,98	1,15	2,58	0,67
**I-TAC / CXCL11**	1,03	0,97	1,34	2,57	5,25
**Presenilin 2**	3,73	9,71	1,08	2,57	1,30
**MAC-1**	9,89	10,37	1,29	2,56	1,26
**HOXD11**	2,63	2,01	1,34	2,53	2,60
**IL-12 p40**	7,09	10,37	1,28	2,53	0,39
**M-CSF R**	8,71	7,62	1,19	2,53	0,65
**VCAM-1 (CD106)**	11,52	6,87	0,72	2,50	0,59
**Tarc**	11,77	10,18	1,07	2,47	0,81
**TRF 2**	6,28	5,75	1,12	2,45	1,31
**PDGF-AA**	7,48	9,25	1,52	2,44	0,82
**SPINK1**	1,53	1,46	2,03	2,43	2,01
**PDGF-BB**	7,99	9,92	1,29	2,43	0,92
**Orosomucoid 2**	7,00	12,77	1,63	2,40	0,55
**GFAP**	9,68	5,98	1,47	2,40	1,87
**SHC1**	4,61	2,89	0,64	2,39	0,61
**JAM-A**	7,49	9,72	1,16	2,39	1,08
**TXNRD2**	0,44	2,60	1,65	2,38	1,76
**VAP-A**	0,97	0,88	1,07	2,34	1,39
**Serpin A7**	4,97	4,13	0,89	2,34	0,48
**TECK / CCL25**	12,98	6,07	1,17	2,33	0,91
**Proteasome 20S alpha**	9,25	1,25	2,42	2,32	2,04
**cIAP-2**	6,13	9,43	1,68	2,32	0,39
**TIMP-2**	1,25	8,37	1,79	2,30	1,78
**TFPI**	5,00	7,22	1,09	2,29	3,76
**Desmoplakin**	7,00	7,71	1,36	2,29	1,20
**TRAIL R3 / TNFRSF10C**	10,25	11,17	0,81	2,27	1,22
**AR (Amphiregulin)**	5,90	14,82	0,82	2,26	2,53
**THOP1**	8,09	5,69	1,00	2,24	0,94
**S100P**	3,26	3,33	0,77	2,24	0,60
**PEPSINOGEN II**	9,90	20,49	1,03	2,23	0,66
**TFF3**	4,12	8,01	1,03	2,22	0,50
**Glycoprotein V**	7,14	5,21	1,23	2,21	1,30
**P-Cadherin**	5,56	10,11	1,14	2,20	1,09
**TIMP-4**	6,29	11,65	0,52	2,15	1,11
**Nestin**	5,43	8,41	0,98	2,15	0,59
**IL-10 R beta**	4,80	4,46	1,55	2,15	1,03
**TGF-beta RIII**	3,20	5,74	0,94	2,15	0,86

GIP	5,96	5,12	1,03	2,14	1,31
ARX	0,87	2,31	1,21	2,12	4,05
BACE-1	6,62	9,45	1,14	2,09	0,56
GSR	2,20	2,08	3,81	2,08	2,39
MMP-3	7,45	6,31	2,15	2,07	1,08
SOX2	0,85	0,96	0,92	2,06	1,00
Zyxin	7,09	4,24	0,89	2,05	3,06
TSH	1,68	1,27	1,93	2,05	1,65
FOLR1	5,49	10,21	1,05	2,05	1,28
LYPA1	2,09	1,42	1,21	2,04	0,57
SIM2	0,69	2,13	1,61	2,03	2,19
Lymphotoxin beta R / TNFRSF3	5,71	8,47	1,06	2,03	0,67
nNOS	5,79	8,94	1,00	2,02	0,49
PlGF	6,91	7,56	1,55	2,02	0,85
MCP-4 / CCL13	6,60	11,54	0,92	2,01	1,62
UQCRH	0,55	0,48	0,55	2,00	0,67
TGF-beta 5	0,47	2,17	1,59	1,99	1,80
Smad 1	0,76	0,79	0,60	1,99	0,61
TNF RII / TNFRSF1B	1,25	2,92	1,40	1,96	2,15
IL-16	5,42	7,20	0,82	1,96	0,67
TMEFF1 / Tomoregulin-1	1,19	0,59	0,63	1,96	1,20
CD35	7,23	7,75	2,91	1,96	4,29
VEGF-D	10,22	5,48	0,73	1,95	0,80
Semaphorin 7A	2,33	0,56	0,98	1,94	0,81
Chem R23	0,24	0,51	0,37	1,93	0,65
DLL1	1,04	0,98	2,13	1,93	1,16
IL-3	3,79	6,14	0,88	1,92	0,43
Proteasome 20S alpha 5	0,60	2,31	1,37	1,91	0,91
SCF	3,89	4,95	1,08	1,90	0,24
BRCA 2	2,97	5,85	0,67	1,90	1,81
HCC-4 / CCL16	2,63	4,09	0,57	1,89	0,51
CRHBP	4,31	7,19	0,87	1,88	1,60
TROPONIN I	4,84	9,20	1,25	1,87	0,33
PTPRZ	0,51	5,78	0,62	1,87	0,59
Pro-MMP-7	4,17	6,82	0,80	1,87	0,30
MIP-1a	0,99	2,48	1,16	1,86	1,19
OSM R beta	2,96	2,77	1,94	1,86	0,84
HSP47	1,51	0,66	0,89	1,86	1,53
Endorepellin	0,95	1,55	1,45	1,85	1,29
Neurofibromin	1,27	4,01	1,15	1,85	1,08
IL-10 R alpha	4,19	4,30	1,32	1,84	0,61
DLL4	1,38	7,28	1,43	1,84	0,98
PARC / CCL18	6,52	6,37	0,85	1,83	0,83
Sonic Hedgehog (Shh N-terminal)	0,42	9,11	0,92	1,83	1,39
MYL12B	1,00	1,58	1,49	1,83	0,84
VEGF R3	4,96	4,39	0,90	1,81	0,64
mGLUR5	5,10	7,33	1,12	1,81	1,18

**GRP**	0,88	0,23	1,93	1,81	0,31
**Coronin 3**	2,67	1,16	0,93	1,80	1,50
**Angiopoietin-4**	5,71	9,64	0,67	1,80	1,44
**Cortactin**	1,35	0,72	1,82	1,80	2,06
**Siglec-5/CD170**	4,14	3,36	1,78	1,80	0,53
**HSP70**	0,91	1,16	1,17	1,80	1,08
**FGFR1**	4,47	6,88	1,14	1,79	0,42
**TCP1 eta**	6,73	1,08	1,44	1,78	1,49
**Desmoglein-2**	5,27	7,87	0,99	1,78	0,89
**LFA-1 alpha**	8,70	9,98	0,92	1,76	1,08
**VE-Cadherin**	4,62	4,72	0,54	1,74	1,29
**CD97**	1,71	0,76	1,54	1,73	1,04
**SERPINB1**	0,94	1,53	1,26	1,72	0,94
**TRAIL R1 / DR4 / TNFRSF10A**	5,49	4,93	0,79	1,71	0,71
**G0/G1switch 2**	0,72	0,55	0,61	1,71	1,16
**Serpin I1**	0,82	8,23	1,20	1,71	1,11
**RPL10**	1,84	1,37	1,31	1,70	1,47
**Reg3A**	1,98	0,88	1,49	1,69	1,05
**PZP**	2,26	0,64	0,65	1,69	0,73
**Fetuin B**	5,66	8,51	0,91	1,68	1,02
**LIGHT / TNFSF14**	4,74	5,38	0,97	1,68	0,44
**RhoGDI**	0,00	1,70	0,21	1,68	1,73
**MIP-1d**	7,04	6,59	0,85	1,67	0,90
**ADAMTS-13**	8,45	6,35	0,75	1,66	1,72
**TIMP-3**	4,83	5,52	0,73	1,66	0,38
**VAP-1**	2,37	1,06	1,41	1,66	1,12
**HGF**	3,95	7,23	1,11	1,66	1,04
**GCP-2 / CXCL6**	4,07	7,79	0,89	1,66	0,74
**Cytokeratin 14**	0,34	0,34	0,34	1,66	0,54
**HAI-2**	4,51	8,73	0,92	1,66	0,86
**Ube2L3**	1,02	1,49	0,37	1,65	2,69
**IP-10**	7,21	10,84	0,97	1,65	1,01
**MBP**	5,31	4,43	0,28	1,63	0,91
**OX40**	0,89	5,64	0,97	1,63	1,13
**RANTES**	5,67	6,39	0,76	1,62	2,17
**Secretogranin V/SCG5**	0,48	1,25	1,08	1,62	0,71
**RAP1AB**	1,19	1,07	1,02	1,62	1,25
**PCPE-1**	0,81	2,98	0,64	1,60	0,52
**beta-Catenin**	0,72	0,28	0,15	1,59	0,89
**Cyclin D1**	2,86	2,94	0,90	1,59	0,75
**Proteasome subunit alpha type 6/PSMA6**	2,29	0,75	0,75	1,58	1,69
**Semaphorin 6B**	5,70	2,27	0,97	1,58	0,54
**UCH-L1**	1,39	0,82	1,42	1,58	0,94
**AGA**	2,32	1,33	3,03	1,58	3,97
**IDH1**	3,91	5,11	0,64	1,57	0,67
**Ck beta 8-1**	2,73	3,25	1,30	1,57	0,87
**Lymphotactin / XCL1**	6,52	6,08	0,69	1,57	0,84

**B7-H3**	3,74	5,14	1,47	1,57	1,32
**DAN**	1,08	2,12	0,93	1,56	2,41
**XIAP**	0,96	0,84	1,68	1,56	0,62
**PGD**	8,86	0,74	0,76	1,55	0,80
**NUP98**	2,48	1,15	1,03	1,55	1,18
**PSMA7**	1,11	0,69	1,14	1,55	1,19
**S100 A8/A9**	4,52	0,52	1,55	1,54	0,33
**MFI2**	4,05	6,84	0,55	1,53	0,97
**Activin B**	2,85	4,75	0,91	1,53	0,45
**OCT3/4**	0,76	12,65	1,57	1,53	1,14
**NGF R**	6,59	5,39	1,35	1,53	1,01
**Orexin A**	6,18	5,20	1,73	1,52	0,84
**ZAP70**	1,18	0,88	2,13	1,52	0,80
**Cadherin-6**	2,82	4,17	0,96	1,52	1,38
**SOD2**	1,42	2,55	1,34	1,51	1,26
**RPS12**	1,12	0,70	1,22	1,51	1,33
**RanGAP1**	1,26	3,07	1,24	1,51	0,87
**Cystatin C**	1,27	0,73	1,27	1,51	0,94
**Osteoactivin / GPNMB**	0,87	0,68	1,32	1,50	1,03
**IGF-I SR**	2,72	4,61	0,63	1,50	0,70
**Hemoglobin A1c**	2,53	1,04	0,44	1,50	0,45
**MSP alpha Chain**	5,14	6,90	1,62	1,49	0,77
**MGP**	13,65	1,77	0,00	1,49	1,08
**Pro-Cathepsin B**	3,88	6,04	0,84	1,48	0,62
**Osteopontin**	0,81	1,40	1,61	1,48	1,20
**LMAN2**	3,45	1,08	1,42	1,48	0,60
**Progesterone**	0,70	1,06	1,29	1,47	0,95
**NT-4**	3,44	3,33	2,27	1,45	0,69
**Uromodulin**	0,60	0,93	0,70	1,44	0,54
**Cadherin-13**	0,64	8,16	0,57	1,44	3,52
**BMP-4**	0,91	1,61	1,84	1,44	1,89
**PARVB**	0,86	2,93	1,57	1,44	1,24
**VIP Receptor 2**	2,65	7,94	0,67	1,43	1,17
**Transferrin**	3,82	13,56	1,51	1,43	2,55
**pro-MMP13**	0,84	0,75	1,36	1,43	1,42
**CNN2**	3,41	0,91	0,65	1,42	1,17
**OSBP1**	6,88	1,47	0,93	1,42	0,55
**RBP4**	3,78	5,67	0,87	1,41	0,54
**MDC**	1,55	1,13	1,69	1,41	1,13
**Lumican**	5,38	4,46	0,94	1,40	0,82
**Brevican**	2,50	3,95	0,86	1,40	0,31
**CD14**	3,07	5,07	0,71	1,39	0,54
**PDLIM1**	0,63	2,11	1,72	1,39	1,65
**RECK**	3,28	4,11	0,78	1,38	0,52
**ARTS1**	2,80	4,25	1,25	1,38	1,34
**PON2**	1,14	5,49	1,49	1,38	0,75
**G-CSF R / CD 114**	3,09	6,56	0,81	1,37	0,74

**NT-3**	3,31	3,00	0,63	1,37	0,27
**Non-muscle Actin/Actin**	0,57	0,86	0,73	1,37	1,35
**RPS2**	0,91	2,34	1,85	1,37	1,38
**Lymphotoxin beta / TNFSF3**	1,05	0,74	1,76	1,36	1,84
**SOX17**	0,40	0,25	0,78	1,36	8,86
**IL-34**	2,75	6,10	1,03	1,36	0,68
**RPS11**	5,39	0,77	0,90	1,36	0,92
**NASP**	1,55	1,18	0,95	1,36	0,98
**LAMP**	3,10	5,96	0,60	1,36	0,70
**TIMP-1**	1,16	0,70	2,97	1,36	1,08
**PSA-total**	4,01	4,67	0,83	1,36	0,96
**Smad 4**	0,60	0,90	1,00	1,36	0,71
**Leptin R**	0,49	0,95	1,79	1,35	1,12
**LIF R alpha**	0,90	0,91	1,65	1,35	1,20
**Kallikrein 14**	3,15	7,40	0,96	1,35	0,78
**DGK**	2,57	3,96	0,61	1,35	0,95
**Cytochrome C**	1,01	0,62	1,36	1,35	1,39
**Serpin B6**	0,53	0,93	0,49	1,34	0,49
**Moesin**	7,08	0,63	0,58	1,34	0,64
**RPL7A**	0,57	0,78	1,02	1,34	1,70
**EphB6**	1,50	1,61	1,10	1,34	1,11
**KRT31**	4,02	0,63	0,54	1,34	0,35
**UBE2D3**	1,66	1,25	0,76	1,33	2,11
**Fibrinopeptide B**	1,23	0,45	0,60	1,33	0,50
**Desmuslin**	1,74	1,71	1,64	1,33	2,08
**ICAM-5**	0,68	1,04	1,23	1,33	0,88
**S100A10**	0,55	0,60	0,37	1,33	0,40
**TAGLN2**	2,45	0,96	1,19	1,33	1,20
**TBCA**	2,06	0,52	1,24	1,33	0,86
**HSP20**	3,17	5,71	0,68	1,32	0,72
**Notch-1**	4,50	4,34	0,84	1,32	0,51
**HB-EGF**	0,84	1,61	1,58	1,32	1,19
**Notch-2**	2,16	2,82	0,89	1,31	0,92
**FACX**	4,58	4,93	0,42	1,31	0,83
**PEDF**	0,87	0,93	1,12	1,31	0,71
**APOA1BP**	2,54	1,46	0,70	1,31	1,39
**SEMA3A**	1,05	1,17	2,03	1,30	1,52
**Serpin B3/SCCA1**	0,73	0,90	1,31	1,30	0,96
**Serpin A10/ZPI**	1,02	0,77	2,58	1,30	1,79
**Syndecan-3**	0,57	9,28	1,79	1,29	1,18
**ACE**	2,90	3,95	1,02	1,29	0,55
**MMP-12**	0,56	0,54	0,47	1,29	0,47
**Plastin L**	0,79	2,06	0,92	1,29	0,78
**PGK-1**	0,48	0,58	0,48	1,29	0,68
**EIF3S2**	2,02	0,50	0,72	1,29	0,73
**HAI-1**	3,79	3,54	0,59	1,29	0,56
**NPM1**	0,68	0,52	0,35	1,28	0,62

HN1	1,09	0,64	1,20	1,28	1,30
Eotaxin / CCL11	5,14	6,65	0,73	1,28	0,60
Tenascin C	1,00	2,97	1,11	1,28	1,08
TLR1	0,60	4,51	0,23	1,27	0,72
Cysteine-rich Protein 1	0,78	0,87	0,67	1,27	1,02
sgp130	2,95	2,64	1,25	1,27	0,44
PPP2R4	2,87	2,98	0,87	1,27	1,17
PCDH7	3,45	1,82	0,89	1,26	1,57
B7-1 /CD80	3,23	4,68	1,13	1,26	0,74
CACNB4	1,92	1,91	2,40	1,26	3,24
Sterol carrier protein 2/SCP2	1,79	0,83	1,28	1,25	1,05
Serpin A11	1,27	0,75	1,56	1,25	1,04
PSMA2	1,12	0,54	1,63	1,25	1,34
Creatine Kinase MM/CKMM	2,12	1,30	1,78	1,25	2,62
RPL10A	3,30	0,78	0,72	1,25	0,97
SCG3	0,52	0,55	0,35	1,25	0,82
ICAM-1	2,64	4,38	0,96	1,25	0,55
LRP-6	0,78	0,67	1,06	1,25	0,72
RELM beta	3,30	3,59	0,61	1,24	0,71
SIGLEC14	2,08	0,86	0,99	1,24	1,40
USP14	1,53	1,19	1,44	1,24	2,19
p53	0,95	0,93	1,29	1,24	1,25
Rab7a	0,65	0,60	0,50	1,24	0,58
Peroxiredoxin 2	0,60	0,50	0,51	1,23	0,55
Perilipin-1	7,10	0,51	0,69	1,22	0,60
Axl	2,35	4,70	0,65	1,22	0,49
PTK 7	0,58	0,58	0,50	1,22	0,58
Integrin beta 6	0,52	0,09	0,79	1,22	0,30
MMR	1,48	4,86	1,08	1,22	1,03
Aldehyde Oxidase 1/AOX1	0,54	0,52	0,55	1,22	0,88
IL-2 R alpha	1,45	1,48	1,18	1,22	1,55
RPL5	3,16	1,16	0,67	1,22	1,21
Ran	0,84	0,88	0,94	1,21	1,03
LDL R	3,58	5,93	1,01	1,21	0,93
14-3-3 gamma	0,65	1,21	1,12	1,21	1,85
SAA	2,45	3,55	0,91	1,21	0,26
SSTR2	0,69	1,18	1,43	1,21	1,26
MPCA	0,80	0,88	0,81	1,21	1,28
Pleckstrin	1,04	1,82	1,06	1,21	1,43
I-309	4,60	6,58	11,47	1,21	3,45
Cardiotrophin-1 / CT-1	7,51	9,49	0,34	1,20	0,43
TWF2	2,20	1,13	0,62	1,20	2,06
Syndecan-1	0,41	0,88	0,31	1,20	0,32
Protein Z	0,63	0,60	0,82	1,20	0,50
SYK	0,55	1,10	1,43	1,20	0,94
Protein C	2,95	1,98	0,89	1,20	0,79
Thrombomodulin	3,11	6,06	1,42	1,20	1,72

**Chymase**	0,66	0,88	1,37	1,20	1,20
**Trypsin 1**	2,82	2,40	0,90	1,20	0,53
**M-CSF**	1,40	1,91	1,48	1,20	1,18
**VEGF**	1,19	0,75	1,46	1,20	0,87
**Epiregulin**	4,16	9,56	1,33	1,20	1,64
**YB1**	1,91	1,08	1,27	1,20	1,78
**RPS3**	1,41	0,71	1,43	1,19	1,12
**Serpin A1**	0,91	1,92	1,73	1,19	1,94
**Afamin**	1,44	0,85	1,33	1,19	1,23
**TRAP1**	1,07	1,00	1,09	1,19	1,93
**Enolase 2**	4,08	4,09	1,09	1,19	0,71
**GITR / TNFRF18**	2,02	1,99	0,78	1,19	0,41
**S100A7**	2,55	2,35	0,66	1,19	0,56
**SPINK7**	1,09	0,98	1,18	1,18	1,30
**RAGE**	4,09	4,43	0,55	1,18	0,41
**SOD-3**	0,64	0,68	0,55	1,18	0,59
**ICAM-2**	0,50	1,04	0,44	1,18	0,93
**Thymidine Kinase-1**	1,03	1,05	1,96	1,18	1,33
**CHC17**	0,75	0,50	0,71	1,17	2,63
**PRDX 1**	3,34	1,71	0,71	1,17	6,61
**WNK2**	3,99	2,13	0,73	1,17	0,55
**perilipin 3**	0,89	0,56	1,19	1,17	0,99
**14-3-3 theta**	1,05	1,10	1,02	1,17	2,79
**TWEAK R / TNFRSF12**	0,61	1,69	0,34	1,17	0,38
**Cathepsin B**	1,28	2,46	1,26	1,17	0,60
**MCM5**	0,49	0,00	1,04	1,17	0,00
**TOPORS**	0,86	1,08	1,50	1,16	1,15
**Legumain**	2,63	3,70	0,59	1,16	0,46
**SynCAM**	1,08	1,55	0,98	1,16	1,79
**Serpin F2**	4,05	0,97	3,81	1,16	1,25
**Ficolin-3**	0,69	0,63	1,32	1,16	1,05
**Vimentin B**	1,39	2,70	1,26	1,16	1,34
**S100A1**	0,31	0,83	0,38	1,15	0,46
**SLITRK1**	3,61	0,90	1,37	1,15	1,00
**Stathmin 1**	1,21	0,63	1,30	1,15	1,12
**EphA1**	0,71	0,78	1,38	1,15	0,85
**Cystatin E/M**	0,95	1,68	1,24	1,15	1,93
**14-3-3 zeta**	0,82	0,65	0,68	1,15	1,06
**Resistin**	0,69	0,38	1,37	1,15	0,87
**SLURP1**	3,14	0,81	1,01	1,15	0,96
**PTP mu**	0,62	0,94	1,29	1,15	1,07
**SHIP**	5,42	0,68	0,88	1,15	0,78
**IGFBP-6**	0,59	0,98	1,43	1,15	0,89
**IGFBP-1**	3,05	3,53	0,96	1,14	0,79
**SUMO3**	2,25	3,70	0,51	1,14	1,17
**ALK**	0,85	1,14	1,24	1,14	1,20
**MANF**	0,45	0,72	0,45	1,14	0,46

eIF4A1-N-t	1,75	0,61	0,35	1,14	1,70
Vitamin D Receptor	0,00	1,82	0,65	1,14	2,16
MMP-25 / MT6-MMP	4,10	4,68	0,82	1,14	1,10
Prolactin	4,15	4,21	0,60	1,14	0,53
FABP5	0,60	0,28	0,79	1,13	1,00
SerRS	3,66	0,82	0,81	1,13	0,75
Cathepsin D	3,25	3,58	0,78	1,13	0,33
TMEM223	1,23	0,74	1,11	1,13	1,04
CD23	0,49	0,68	0,63	1,13	1,07
EpCAM	0,62	0,50	1,30	1,13	1,26
Uteroglobin(1)	1,42	1,84	1,02	1,13	0,78
PCBP2	6,25	0,55	0,65	1,13	0,59
RPS19	6,74	0,45	0,80	1,13	0,97
hnRNP C1 + C2	2,43	3,37	0,74	1,12	0,91
MICB	0,91	0,84	1,54	1,12	0,74
MMP-15	3,34	2,74	0,69	1,12	0,57
Soggy-1	3,49	3,49	2,40	1,12	0,48
CTGF / CCN2	2,71	5,48	0,52	1,12	0,31
UQCRB	1,30	0,87	0,96	1,12	1,10
FGFR2	3,04	4,36	0,71	1,12	0,26
ROR2	3,84	6,04	0,60	1,12	0,91
PON1	0,56	6,30	1,07	1,12	0,74
FAP	2,27	2,81	0,91	1,11	0,31
TPA	1,16	2,78	0,66	1,11	0,31
hnRNP M1-M4	1,43	1,67	2,23	1,11	2,35
IL-13 R alpha 2	2,44	3,14	0,64	1,11	0,41
IGFBP-7 (IGFBP-rp1)	3,48	5,93	1,52	1,11	1,23
PDGF-D	0,35	0,42	0,34	1,11	0,30
Symplekin	5,00	2,35	1,68	1,11	0,69
RPS23	2,18	0,82	1,33	1,10	0,99
Lefty - A	0,99	0,69	1,27	1,10	1,10
CTACK / CCL27	0,86	1,36	1,27	1,10	1,51
NCAM2	4,93	0,84	0,75	1,10	0,75
SLC38A10	4,35	0,71	0,98	1,10	0,88
eIF5A	0,51	1,21	0,56	1,10	1,15
INSL3	0,83	1,33	0,83	1,10	1,41
Kallikrein 8	0,82	0,53	1,28	1,10	0,82
TACI / TNFRSF13B	2,33	2,10	0,54	1,09	0,50
Wilms Tumor 1	0,96	0,54	2,40	1,09	0,95
LOX-1	3,34	4,78	0,50	1,09	0,31
Beta IG-H3	2,13	2,45	1,50	1,09	0,87
PDGF R alpha	1,88	1,88	0,53	1,09	0,32
Frizzled-3	0,48	6,92	1,09	1,09	1,06
SMC4	0,78	0,66	0,97	1,09	0,79
SDF4	1,35	1,32	1,03	1,08	1,36
COCO	8,46	4,74	0,74	1,08	0,56
GART	1,75	0,31	0,92	1,08	0,53

SH3BGRL3	8,54	0,44	0,74	1,08	0,77
SDNSF	10,41	0,86	0,30	1,08	0,27
TROY / TNFRSF19	1,11	1,66	0,57	1,08	0,35
Tenascin X(1)	2,56	0,90	0,93	1,08	1,12
P4HB	0,83	0,71	1,05	1,07	0,79
TIM-1	0,54	0,97	0,43	1,07	0,31
Kallikrein 5	0,99	3,38	1,17	1,07	4,86
CD47	2,09	2,22	1,46	1,07	0,75
TRPM7	1,07	0,74	1,17	1,07	0,89
Neg	1,75	0,66	0,86	1,07	2,39
TPM4	0,71	1,51	1,69	1,07	1,04
NAP-2	2,64	3,50	0,62	1,07	0,42
E-Cadherin	1,22	1,08	1,41	1,06	1,17
EEF2	1,16	0,50	1,09	1,06	1,06
Calbindin D	0,95	2,10	0,88	1,06	1,42
IFRD1	5,01	0,64	2,45	1,06	0,72
VNN1	1,20	1,53	1,33	1,06	2,17
IL-1 beta	2,36	2,41	0,78	1,06	0,58
Gephyrin	3,64	2,01	0,66	1,06	1,04
Phosphoserine Aminotransferase/PSAT1	2,28	1,62	2,31	1,05	0,96
ZC3H4-N-t	1,36	0,95	0,76	1,05	1,85
Annexin A2	1,88	3,98	0,50	1,05	1,16
Notch-2 ICD	0,50	4,71	0,66	1,05	0,87
IL-17C	0,94	1,12	1,53	1,05	0,90
Pref-1	0,47	0,75	2,17	1,05	0,90
TXNDC4	1,47	1,17	1,24	1,05	1,55
MMP-1	3,62	4,42	0,56	1,05	0,37
S100A9	2,94	0,95	1,51	1,05	1,08
IL-22 BP	3,02	4,30	0,63	1,04	0,38
CETP	2,25	2,67	3,01	1,04	4,95
DEFA1/3	1,95	1,23	0,99	1,04	1,21
MIP-1b	0,74	0,99	1,34	1,04	0,84
RCL	2,07	0,73	1,32	1,04	1,45
Cux2	1,68	0,74	0,45	1,04	0,60
SF20	0,40	0,61	0,27	1,04	0,42
MICA	3,09	2,61	0,71	1,04	0,46
Adipsin	2,70	3,17	0,67	1,03	0,50
NR3C3	1,10	1,19	1,19	1,03	1,29
MIP-3 alpha	2,53	4,74	0,63	1,03	2,38
Annexin A6	1,44	3,45	0,14	1,03	0,98
TSLP	0,49	1,02	0,42	1,03	0,32
Dkk-1	1,87	3,13	0,48	1,03	0,60
SART1	1,06	1,36	0,85	1,03	1,10
TCEB2	5,38	0,60	0,91	1,03	0,81
Salivary alpha amylase/aAmylase	5,70	0,54	0,73	1,03	0,58
CD200	3,02	3,99	1,16	1,03	1,05
MIF	2,81	2,93	0,77	1,02	0,60

**Pentraxin3 / TSG-14**	0,38	0,60	0,31	1,02	0,30
**MLCK**	5,85	0,64	0,66	1,02	0,65
**CD42b**	1,59	2,29	1,11	1,02	0,52
**Activin RIIA**	1,42	1,80	1,83	1,02	1,98
**Grainyhead-like protein 1 homolog/GRHL1**	0,80	1,11	0,70	1,02	1,28
**FAM3C**	2,03	2,29	0,47	1,02	0,58
**Podocalyxin**	1,02	1,04	1,34	1,02	1,04
**HSP90**	0,93	1,50	0,93	1,02	0,93
**C1qR1**	1,76	3,14	1,10	1,02	1,30
**PREP**	0,59	0,42	0,41	1,02	0,53
**HINT1**	4,82	0,63	0,62	1,02	0,79
**p27**	0,68	0,56	1,17	1,02	0,88
**Osteoprotegerin / TNFRSF11B**	1,38	1,03	1,84	1,02	1,13
**ApoE**	0,95	3,25	1,31	1,01	1,15
**Apo (a)**	3,08	0,56	1,14	1,01	1,55
**Alpha 1 Microglobulin**	1,87	0,81	0,89	1,01	1,05
**beta 1 Spectrin**	1,78	1,25	1,13	1,01	1,25
**hCGb**	3,50	3,84	0,74	1,01	0,56
**Orexin B**	2,06	1,55	1,30	1,01	0,49
**Vasorin**	2,05	2,82	1,01	1,01	0,85
**ADAM-9**	0,64	0,59	0,74	1,01	0,46
**CRIM 1**	2,17	3,04	0,75	1,01	0,52
**RNASE6**	8,67	0,65	0,76	1,01	0,58
**ERAB**	1,36	0,61	0,66	1,01	0,74
**SHANK1**	1,34	0,90	1,34	1,01	1,11
**Angiopoietin-like 2**	1,07	0,92	1,27	1,01	2,27
**Kallikrein 6**	0,93	1,70	0,88	1,01	1,54
**TOB2**	1,43	0,42	0,86	1,01	0,78
**Titin**	1,07	0,88	1,23	1,00	1,27
**Glut3**	0,42	0,22	0,50	1,00	1,52
**ERAP2**	1,66	2,75	1,35	1,00	1,42
**DPEP2**	0,00	0,30	1,29	1,00	1,42
**Smad 8**	0,27	0,98	0,47	1,00	0,39
**CA2**	0,72	2,18	0,59	1,00	1,23
**BAI-1**	1,84	1,17	0,79	1,00	0,42
**Serpin A5**	0,85	1,47	0,69	1,00	1,16
**Growth Hormone (GH)**	1,56	2,55	1,33	1,00	1,03
**IL-18 R alpha /IL-1 R5**	1,85	1,91	0,62	1,00	0,24
**IL-1 F8 / FIL1 eta**	1,94	2,68	1,11	1,00	1,10
**Claudin-3**	0,80	1,00	1,30	0,99	1,22
**Noelin**	0,88	1,18	1,18	0,99	1,36
**PYGL**	8,42	0,41	0,75	0,99	0,68
**FH**	0,96	0,99	0,79	0,99	1,70
**Calpain S1**	1,34	0,33	0,50	0,99	0,49
**Activin A**	1,79	3,29	0,78	0,99	0,44
**hHR23b**	3,55	0,86	1,22	0,99	1,23
**UPB1**	1,13	0,94	0,96	0,99	1,38

**Calreticulin**	0,84	0,48	1,01	0,99	0,80
**14-3-3 sigma**	3,13	1,60	2,52	0,99	4,76
**Siglec-9**	1,25	1,23	1,18	0,99	1,40
**RIP1**	0,80	1,08	0,51	0,99	0,40
**PCBP1**	0,79	1,26	1,16	0,99	1,09
**Erythropoietin R**	0,70	1,10	1,34	0,99	1,10
**NAP1L1**	3,28	0,55	0,77	0,98	0,48
**Smad 7**	0,27	0,23	0,24	0,98	0,16
**Laminin 2 alpha**	1,22	0,92	1,19	0,98	1,12
**Metavinculin**	0,94	0,70	1,19	0,98	1,04
**Cystatin A**	0,68	0,86	1,22	0,98	1,28
**GRP**	6,75	0,53	0,88	0,98	1,12
**PCYOX1**	5,54	0,75	1,20	0,98	1,79
**TFF2(1)**	2,39	1,05	1,37	0,98	0,92
**WISP-1 / CCN4**	0,68	1,12	0,40	0,98	0,41
**IL-4**	1,83	2,50	0,48	0,98	0,37
**GLRX1**	0,55	0,56	0,79	0,98	0,70
**Neurabin 1**	5,95	0,52	0,79	0,98	0,52
**SOD1**	1,01	1,11	1,01	0,97	0,56
**NAPRT1**	0,90	0,43	0,38	0,97	0,33
**NF1**	1,11	2,37	1,19	0,97	2,08
**QDPR**	0,49	0,62	0,37	0,97	0,36
**CD61**	0,76	1,10	1,29	0,97	1,01
**hnRNP G**	4,27	1,00	0,77	0,97	0,86
**IL-1 ra**	0,69	1,39	1,17	0,97	0,98
**GATA-3**	0,30	0,88	0,33	0,97	0,45
**GRO-a**	1,16	1,51	1,63	0,97	1,55
**HNF-3 alpha /FoxA1**	6,72	6,77	1,89	0,97	2,16
**NeuroD1**	0,31	0,27	0,29	0,97	0,30
**Survivin**	2,77	4,05	0,99	0,97	0,71
**Neuritin**	0,82	0,84	1,42	0,96	0,78
**Glyoxalase II**	0,45	0,62	0,45	0,96	0,74
**BMP-3b / GDF-10**	0,39	0,69	0,18	0,96	0,64
**GPI**	1,14	1,74	1,09	0,96	1,48
**Plectin**	0,76	0,59	0,72	0,96	0,98
**VDAC1 / Porin**	1,14	0,89	1,43	0,96	1,12
**GDNF**	2,75	4,75	0,66	0,96	1,16
**Cathepsin G**	1,49	1,24	1,61	0,96	2,95
**Versican isoform V0**	1,02	0,77	0,75	0,96	0,79
**Dtk**	2,32	3,05	0,67	0,96	0,59
**ALDH1A1**	1,07	0,95	1,94	0,96	2,58
**alpha -Synuclein**	1,16	1,00	1,29	0,96	1,15
**FASN**	0,78	0,18	0,94	0,96	0,85
**CA 9**	0,95	0,59	1,16	0,96	0,80
**MTUS1**	2,94	3,52	0,60	0,96	0,36
**6Ckine**	1,16	2,02	0,64	0,96	0,31
**IL-22**	0,88	2,10	1,62	0,96	2,14

**ROCK1**	2,51	3,49	0,59	0,96	0,19
**GPX3**	0,80	1,77	0,45	0,95	0,42
**RPS25**	5,01	0,67	0,45	0,95	0,72
**11b-HSD1**	0,51	0,83	0,43	0,95	0,50
**IL-31**	0,31	0,52	0,27	0,95	0,16
**TrypsinPan**	5,02	0,56	0,84	0,95	0,56
**IGFBP-2**	1,88	2,65	0,78	0,95	0,45
**PSMC3**	0,75	0,66	0,96	0,95	0,62
**PDLIM5**	0,93	1,43	0,35	0,95	1,00
**SCF R /CD117**	2,69	2,19	0,67	0,95	0,27
**SEZ6L2**	0,34	0,59	0,32	0,94	0,37
**Hemopexin**	2,70	2,46	0,94	0,94	0,48
**Src(1)**	3,08	0,73	1,26	0,94	0,90
**SAMSN1**	1,03	1,26	0,98	0,94	1,05
**D4 GDI**	1,05	0,32	0,76	0,94	0,48
**OX40 Ligand / TNFSF4**	2,22	2,53	1,06	0,94	0,71
**Fas / TNFRSF6**	1,66	9,23	0,86	0,94	0,30
**BCAM**	1,05	0,80	1,13	0,94	1,96
**FABP1**	0,76	0,98	1,22	0,94	0,95
**14-3-3 eta**	1,04	0,80	0,81	0,93	1,51
**IL-29**	1,00	1,18	1,14	0,93	0,95
**Apolipoprotein L 2**	1,51	1,82	1,06	0,93	2,60
**Periostin**	0,63	0,81	0,75	0,93	0,59
**SP-D**	2,75	1,00	2,18	0,93	0,92
**NEP**	0,45	0,58	0,38	0,93	0,40
**Midkine**	0,62	0,65	0,61	0,93	0,47
**Serpin B8**	0,50	0,60	0,46	0,93	0,48
**CD90**	0,94	1,07	1,43	0,93	1,20
**BMP-6**	0,95	0,82	1,46	0,93	1,10
**PDZD2**	1,73	0,83	1,08	0,93	0,70
**ALCAM**	2,13	2,95	0,50	0,93	0,47
**G3BP**	2,37	0,55	0,65	0,93	0,75
**PD-ECGF**	2,66	3,36	0,42	0,93	0,35
**VEGF-C**	5,41	0,72	0,79	0,92	0,45
**RKIP**	0,48	0,72	0,39	0,92	0,34
**SSTR5**	3,83	4,63	0,66	0,92	0,50
**Cathepsin L**	0,39	0,78	0,36	0,92	0,36
**EPHX2**	1,58	1,18	0,56	0,92	1,10
**Triosephosphate isomerase/TPIS**	0,98	1,45	1,28	0,92	1,22
**RPL12**	2,16	0,98	1,08	0,92	0,83
**MAN1**	1,69	0,95	0,88	0,92	0,53
**Lipocalin-1**	0,97	0,80	1,53	0,92	0,94
**MCM**	4,61	2,45	1,03	0,92	2,11
**TRAIL R2 / DR5 / TNFRSF10B**	1,19	2,00	1,24	0,92	1,08
**Ribonuclease Inhibitor**	6,92	0,35	0,37	0,92	0,51
**TGF-alpha**	0,49	0,78	0,30	0,92	0,34
**ACACA**	0,52	0,43	0,70	0,92	0,88

**LIF**	3,54	1,11	1,05	0,91	1,01
**Proteasome subunit beta type 4/PSB4**	0,53	0,97	0,75	0,91	0,76
**FABP4**	0,47	1,13	0,68	0,91	0,49
**SOD4**	0,71	1,34	1,21	0,91	0,66
**SUCLG1**	4,21	0,70	0,89	0,91	0,86
**Transketolase/TALDO**	3,10	1,48	1,28	0,91	0,41
**SREC-II**	0,88	0,83	1,04	0,91	0,69
**Nidgen-1**	1,54	2,62	0,90	0,91	0,54
**Cerberus 1**	0,42	0,91	1,16	0,91	1,09
**SPTBN1**	0,88	0,92	1,01	0,91	1,08
**TLR4**	0,91	0,60	0,92	0,91	0,63
**IL-19**	0,83	1,00	1,08	0,91	0,90
**Proteasome subunit beta type 2/PSB2**	0,57	0,93	0,86	0,91	0,73
**MMP-11 /Stromelysin-3**	1,33	0,48	1,13	0,91	0,68
**EPCR**	0,56	1,75	1,69	0,91	2,77
**ITM2B**	0,46	0,62	0,49	0,91	0,56
**DDX3Y**	1,35	1,00	0,97	0,91	1,50
**ADAMTS-5**	0,79	0,47	1,29	0,90	0,68
**YY1**	1,89	0,68	1,13	0,90	0,92
**CPS1**	1,21	0,46	0,58	0,90	0,58
**IL-31 RA**	0,46	0,56	0,30	0,90	0,26
**IL-2 R gamma**	2,27	2,55	0,73	0,90	0,59
**BPIFB1**	2,73	0,73	2,00	0,90	0,46
**SHP-1**	0,83	0,72	0,69	0,90	0,66
**APRIL**	0,93	1,68	0,85	0,90	1,64
**ACAA2**	1,25	0,64	0,78	0,90	0,65
**PRSS23**	1,87	0,50	0,62	0,90	0,57
**RELT / TNFRSF19L**	1,12	0,76	1,49	0,89	0,83
**CA3**	2,02	0,79	0,67	0,89	0,66
**E-Selectin**	1,38	2,01	0,74	0,89	0,39
**E1 Ubiquitin Activating Enzyme/UBA1**	1,26	0,27	0,42	0,89	0,35
**CA150**	2,12	0,58	1,64	0,89	2,98
**Furin**	3,38	4,15	0,53	0,89	0,63
**CD155**	2,93	3,89	1,88	0,89	1,61
**UNC-13 Homolog D**	2,15	0,76	0,92	0,89	1,13
**IL-2 R beta /CD122**	1,87	2,07	0,67	0,89	0,58
**PTN**	0,51	0,49	0,35	0,89	0,67
**LAMA**	0,49	0,59	0,31	0,89	0,51
**WDR1**	1,22	0,96	0,76	0,89	2,00
**PAK7**	1,29	1,16	1,32	0,89	1,08
**PTP kappa**	2,37	0,93	1,14	0,88	0,71
**Osteoadherin(2)**	1,06	0,69	1,03	0,88	1,07
**Ubiquitin+1**	0,90	0,46	1,32	0,88	0,62
**ENA-78**	1,68	2,89	0,45	0,88	0,15
**Nanog**	0,45	0,68	0,49	0,88	0,42
**MCP-2**	2,30	5,66	0,21	0,88	0,53
**VEGI / TNFSF15**	0,94	1,04	0,40	0,88	0,79

**Alpha Fodrin**	1,29	1,45	0,20	0,88	1,59
**IL-33**	0,60	0,89	0,46	0,88	0,34
**PCK2**	5,99	0,49	0,72	0,88	0,34
**TXNDC5**	1,11	0,94	0,90	0,88	0,93
**BTF3**	0,47	1,00	0,33	0,88	1,19
**IL-17D**	0,88	0,48	1,14	0,87	0,66
**PARK7**	1,34	1,13	1,07	0,87	1,14
**Csk**	1,78	0,42	0,12	0,87	0,29
**Lamin B2**	6,86	0,40	0,49	0,87	0,46
**Metallothionein**	0,82	2,53	0,80	0,87	0,31
**NM23-H1/H2**	0,47	1,20	0,34	0,87	0,65
**Myoglobin**	0,62	0,76	1,08	0,87	0,92
**FGF R3**	1,83	2,54	0,61	0,87	0,52
**Karyopherin beta 1**	0,81	0,56	1,49	0,87	0,56
**FoxO1**	0,54	0,70	0,43	0,87	0,66
**RPL11**	0,81	1,07	0,85	0,87	0,78
**Serpin A9**	0,55	0,59	0,40	0,87	0,52
**CCL14 / HCC-1 / HCC-3**	0,51	3,43	0,94	0,87	0,55
**Brg1**	0,47	0,63	0,58	0,87	0,71
**Peroxiredoxin 3**	1,87	1,60	0,83	0,86	0,69
**AMICA**	0,40	1,11	0,21	0,86	0,32
**ACE-2**	0,99	0,85	1,29	0,86	1,05
**IFN-beta**	1,90	1,85	0,66	0,86	0,48
**HABP2**	4,24	0,50	1,14	0,86	1,16
**ARP19**	1,47	0,14	0,98	0,86	1,24
**Ferritin**	2,55	3,40	0,90	0,86	0,40
**2B4**	0,53	0,85	0,18	0,86	0,39
**Vitamin D-BP**	0,56	0,82	1,17	0,86	0,72
**HGFR**	0,60	0,91	0,57	0,86	0,50
**Serpin A8**	0,52	0,63	0,50	0,86	0,31
**GALNT2**	0,51	0,77	0,58	0,86	0,48
**Prohibitin**	0,55	0,70	0,41	0,86	0,42
**Peroxiredoxin 5**	0,49	1,19	0,42	0,86	0,46
**BASP1**	1,88	0,50	0,53	0,86	0,48
**Collagen I a1**	0,62	0,99	0,79	0,85	0,60
**p16 ARC**	6,43	0,49	0,70	0,85	0,56
**Gas1**	2,85	3,70	0,53	0,85	0,84
**Biglycan**	2,60	1,91	0,83	0,85	0,36
**Osteocalcin**	3,42	3,00	0,71	0,85	0,35
**Prosaposin**	5,93	0,27	0,41	0,85	0,09
**LIN41**	0,49	0,70	0,37	0,85	0,42
**DAK**	1,36	1,18	1,95	0,85	1,68
**NIT2**	6,71	0,61	0,58	0,85	0,80
**Serpin A3**	0,58	1,75	0,93	0,85	1,71
**IL-18 BPa**	1,68	1,22	1,45	0,85	0,40
**MMP-7**	0,96	0,59	1,82	0,85	1,20
**VEGF-B**	0,68	1,60	0,21	0,85	0,37

**PCAF**	0,59	0,85	1,33	0,85	0,80
**Livin**	0,40	0,73	0,37	0,85	0,32
**DEP-1**	1,69	1,70	1,04	0,84	0,86
**Tropomyosin 3**	0,81	0,97	1,71	0,84	1,03
**TGF-beta 2**	1,18	1,18	1,44	0,84	1,19
**Prion protein PrP /PRNP**	5,14	1,20	0,44	0,84	0,41
**TRANCE**	0,58	0,58	1,03	0,84	2,64
**RNA Polymerase II/POLR2A**	0,39	0,42	0,36	0,84	0,28
**Angiopoietin-2**	0,51	0,76	0,82	0,84	0,80
**Neg**	1,49	3,93	1,05	0,84	1,31
**PD-1**	0,43	0,91	0,37	0,84	0,38
**PGAM1**	0,89	0,98	1,52	0,84	0,58
**Smad 5**	1,74	1,73	3,14	0,84	2,36
**SAA4a**	0,52	0,63	0,64	0,84	0,43
**Desmocollin-3**	0,41	1,00	0,37	0,84	0,64
**ProSAAS**	0,81	0,88	1,19	0,84	1,06
**RPL14**	0,58	1,19	1,00	0,84	0,78
**CDC5L**	1,64	0,78	0,70	0,83	0,88
**B4GalT1**	0,89	0,65	1,22	0,83	3,07
**PIM2**	0,73	0,60	1,22	0,83	0,98
**CCR7**	0,82	1,24	1,02	0,83	1,26
**PCNA**	0,77	0,92	1,13	0,83	0,85
**CD55**	1,06	0,70	1,43	0,83	0,72
**alpha 1,2 Mannosidase IA**	1,01	0,37	1,23	0,83	0,86
**ROR1**	1,87	2,43	0,94	0,83	0,75
**ORP150**	0,71	2,44	1,40	0,83	0,83
**ZNF671**	1,13	1,09	1,18	0,83	1,00
**4-1BB**	0,42	0,59	0,48	0,83	0,28
**ACLP**	1,15	1,68	0,88	0,83	0,59
**B3GNT2**	0,99	0,57	0,68	0,83	0,87
**ADAMTS-4**	0,91	1,11	1,26	0,83	0,96
**PSP**	1,99	0,50	0,65	0,83	0,29
**RANK / TNFRSF11A**	4,28	1,34	0,98	0,83	0,59
**IL-7 R alpha**	1,56	1,22	0,59	0,83	0,43
**GULP1/CED-6**	0,68	0,59	0,95	0,82	1,14
**Latent TGF-beta bp1**	2,14	2,48	0,73	0,82	0,51
**MMP-2**	1,78	1,19	1,20	0,82	1,06
**IGSF4B**	0,96	2,20	1,13	0,82	0,89
**DOT1L**	0,93	0,77	1,24	0,82	0,96
**ECM-1**	1,46	2,30	1,26	0,82	1,57
**MAGI2**	0,69	0,53	0,44	0,82	0,54
**DSPG3**	2,87	3,76	0,55	0,82	0,96
**H6PD**	6,17	0,74	1,02	0,82	1,23
**OSCAR**	0,65	0,42	0,84	0,82	0,72
**Cystatin SN**	0,87	1,19	1,43	0,82	1,57
**IL-6 R**	1,14	3,22	1,56	0,82	0,67
**TLS/FUS**	0,76	0,60	1,05	0,82	1,24

**FLRG**	0,50	2,35	0,69	0,81	0,49
**Kallikrein 7**	0,70	0,83	1,53	0,81	0,84
**FABP2**	2,53	3,30	0,89	0,81	0,74
**P-selectin**	0,80	0,76	1,17	0,81	0,54
**BMP-7**	2,60	5,13	0,89	0,81	0,70
**C2**	0,40	0,51	0,55	0,81	0,48
**Proteasome 26S S5**	0,41	0,75	0,86	0,81	1,09
**Beta 2M**	0,65	2,16	0,36	0,81	0,35
**EphA6**	1,94	0,36	0,85	0,81	0,34
**TRPC1**	0,59	0,70	0,47	0,81	0,29
**14-3-3 epsilon**	0,82	0,87	0,89	0,81	1,53
**HSP60**	0,85	0,61	1,54	0,81	1,01
**DANCE**	2,64	4,66	0,37	0,81	0,37
**CXCR1 / IL-8 RA**	1,19	1,26	1,70	0,81	1,80
**ApoD**	1,60	0,39	0,65	0,81	0,39
**Aggrecan**	1,10	0,75	1,02	0,81	1,39
**CD39L4**	1,27	1,03	1,36	0,80	1,65
**Cytokeratin 8**	3,25	4,16	1,19	0,80	1,06
**Galectin-7**	0,40	1,17	0,42	0,80	0,40
**ERp57**	0,48	0,51	0,53	0,80	0,74
**NET1**	2,98	0,67	0,51	0,80	0,54
**CEACAM-8/CD66b**	1,48	1,80	0,95	0,80	1,39
**IL-18 R beta /AcPL**	1,91	2,21	0,73	0,80	0,65
**S100A4**	0,57	0,64	0,55	0,80	0,59
**ADAMTS-L2**	0,64	0,13	0,44	0,80	0,08
**PDX-1**	0,61	0,79	1,14	0,80	1,02
**PPP2R1B**	0,61	1,96	0,97	0,80	2,63
**Cystatin S**	0,90	1,22	1,28	0,80	1,32
**PLA2G1B**	1,79	1,12	1,55	0,80	1,03
**SOST**	1,08	0,89	1,16	0,80	0,95
**GDI1**	1,61	0,26	0,32	0,80	0,43
**MP1**	0,52	0,84	1,00	0,80	1,25
**Reg1A**	2,61	0,82	0,98	0,80	0,70
**URB2**	0,23	1,96	0,81	0,80	0,81
**LMW-PTP/ACP1**	0,34	0,63	0,39	0,80	0,12
**ROS**	2,43	1,02	1,36	0,80	0,74
**PRCP**	0,76	0,49	0,55	0,79	0,33
**TRA-1-81**	0,83	1,25	1,67	0,79	0,70
**14-3-3 beta**	0,58	0,47	0,54	0,79	0,70
**FUCA2**	1,34	0,88	2,18	0,79	3,23
**LECT2**	0,64	0,83	0,48	0,79	0,49
**Fen 1**	1,45	1,42	0,58	0,79	0,31
**PNP**	0,46	0,41	0,60	0,79	0,39
**CRTAM**	0,53	0,80	0,42	0,79	0,37
**SorLA**	0,91	0,94	0,45	0,79	1,00
**LIMPII**	0,52	0,84	0,35	0,79	0,49
**USP5**	0,39	1,26	0,51	0,79	0,55

IGF-I	1,57	2,62	0,54	0,79	0,57
Amylin	0,64	0,51	0,57	0,79	0,42
CAPZA1	1,12	0,53	0,41	0,79	0,42
SIGIRR	0,71	1,22	1,14	0,79	1,00
Dystroglycan	0,52	0,95	0,64	0,78	0,79
Neural Cadherin	0,53	0,47	0,66	0,78	0,87
Myoferlin	5,44	0,44	0,61	0,78	0,37
ADM	1,44	2,83	0,74	0,78	0,75
Kremen-1	1,24	1,87	0,90	0,78	0,55
FSH	1,64	0,62	0,94	0,78	0,63
Lamin A + C	6,52	0,36	0,18	0,78	0,52
TRAIL / TNFSF10	1,30	1,27	2,31	0,78	1,11
RPS20	0,43	1,23	0,66	0,78	0,39
Guanylin	4,91	0,17	0,65	0,78	0,84
Trypsinogeb-2	1,77	0,66	1,39	0,78	0,84
NF-M	1,95	3,46	0,46	0,78	0,55
Ras	0,51	1,07	1,19	0,78	0,92
Pro-BDNF	0,78	0,73	1,43	0,78	0,98
IL-17E	1,71	2,33	0,62	0,77	0,52
PTH	0,41	0,34	1,11	0,77	1,08
HVEM / TNFRSF14	2,01	4,37	0,67	0,77	0,68
Ribonuclease T2	2,80	0,75	0,90	0,77	1,17
Nucleobindin 1/NUCB1	0,85	0,65	0,79	0,77	1,07
PEPD	3,49	0,47	0,69	0,77	0,42
SerpinE2	2,57	0,83	1,41	0,77	0,93
Serpin B5	1,49	0,98	1,60	0,77	1,14
C5b-9	2,23	0,32	1,16	0,77	1,37
ZAG	0,66	0,98	1,20	0,77	0,63
ERp29	0,51	0,58	0,61	0,77	0,66
LAG-3	0,62	0,86	0,49	0,77	0,58
Bassoon	0,87	1,00	1,07	0,77	0,91
GLUD1	1,17	0,10	0,20	0,77	0,55
FGF-7 / KGF	1,70	2,16	0,48	0,77	0,26
Glypican 5	0,87	0,76	0,94	0,77	0,74
Cytokeratin 9	1,01	0,19	0,42	0,77	0,42
NAGLU	5,44	0,46	0,59	0,76	0,48
BMP-1	0,90	1,23	1,38	0,76	1,46
GLO-1	0,27	0,80	0,30	0,76	0,20
CD5L	0,67	0,41	0,75	0,76	0,59
ACTH	1,16	1,56	0,86	0,76	0,56
GPBB	1,43	0,87	1,29	0,76	1,19
Cytokeratin 15	3,88	1,98	2,61	0,76	3,97
CEACAM-1	0,77	1,54	1,14	0,76	1,05
EGF	1,42	3,00	0,60	0,76	0,39
NrCAM	0,68	1,58	0,91	0,76	1,36
NEDD8	0,34	0,56	0,35	0,76	0,35
CNOT1	0,82	0,11	1,25	0,76	0,81

**GMF beta**	1,00	0,72	1,29	0,76	0,65
**TRPC6**	0,93	0,49	0,97	0,76	0,73
**IL-1 F7 / FIL1 zeta**	0,97	0,76	1,52	0,76	0,76
**CXCR3**	2,15	3,51	0,61	0,76	0,67
**Thrombospondin-4**	1,28	2,55	1,25	0,76	1,20
**GM2A**	1,19	0,62	1,26	0,76	1,10
**ADAMTS-10**	2,00	4,17	0,78	0,76	0,49
**Contactin-2**	2,28	2,59	0,85	0,75	0,67
**LYRIC**	3,16	1,39	0,64	0,75	2,33
**FASTKD5**	2,06	0,43	0,40	0,75	0,61
**NPTXR**	0,39	0,95	0,41	0,75	0,34
**Chitotriosidase**	0,63	0,84	0,41	0,75	0,75
**OSM**	0,64	0,58	0,89	0,75	0,73
**FGF-BP**	1,18	1,60	0,66	0,75	0,69
**ALS**	0,85	0,54	1,01	0,75	1,84
**Galectin-3BP**	0,69	0,55	0,68	0,75	0,47
**Galectin-1**	0,73	0,85	0,40	0,75	0,41
**PKM2**	0,27	0,68	0,36	0,75	0,43
**Pancreatic Polypeptide**	0,58	0,95	1,28	0,75	1,00
**beta-NGF**	1,75	3,10	0,38	0,75	0,36
**Serpin A4**	1,07	1,24	0,85	0,75	0,30
**AlphaA Crystallin/CRYAA**	0,66	0,64	0,82	0,75	0,97
**ApoC2**	0,73	0,83	0,66	0,75	0,66
**IL-24**	0,77	0,76	1,22	0,75	0,82
**Mesothelin**	0,42	4,12	0,72	0,75	0,73
**FoxP3**	0,49	0,82	0,42	0,75	0,36
**LRP-1**	0,87	0,69	0,44	0,75	0,56
**EphB4**	0,96	1,31	0,46	0,75	0,62
**CFHR2**	1,84	2,15	0,73	0,75	0,44
**ApoE3**	0,31	0,59	0,27	0,75	0,26
**hCG alpha**	1,87	1,41	1,14	0,75	0,79
**KMT2B**	1,35	0,89	1,03	0,74	0,69
**PECAM-1 /CD31**	2,72	2,04	0,55	0,74	0,65
**FGF-23**	0,55	0,76	0,91	0,74	0,95
**S100A11**	0,70	0,74	1,43	0,74	1,39
**IL-1 F5 / FIL1delta**	1,21	1,57	1,36	0,74	0,97
**Attractin**	0,60	0,70	1,10	0,74	0,40
**HEG1**	7,67	0,21	0,75	0,74	1,31
**Persephin**	0,25	0,19	0,25	0,74	0,16
**DPPI**	1,75	0,64	2,15	0,74	2,49
**AGXT**	1,24	0,43	0,68	0,74	0,39
**CPN1**	1,16	0,41	0,62	0,74	0,44
**HMGB1**	0,95	0,27	0,84	0,74	1,46
**beta -I Tubulin**	0,63	1,02	1,61	0,74	1,23
**GRP78**	1,01	0,90	1,09	0,73	1,19
**SHBG**	1,42	0,81	0,91	0,73	0,26
**BAZ2B**	0,75	1,27	0,83	0,73	1,02

**Cyclophilin B**	0,04	0,79	0,45	0,73	0,55
**IL-1 sRI**	1,42	2,67	0,25	0,73	0,30
**BLMH**	0,56	0,81	0,64	0,73	0,68
**Calcitonin**	1,88	0,44	0,83	0,73	0,35
**Fibrinogen**	0,81	0,91	1,27	0,73	1,05
**HSP10**	0,58	0,80	0,97	0,73	0,96
**Somatostatin**	2,69	0,82	1,48	0,73	1,00
**FGF-18**	1,69	0,44	0,47	0,73	0,29
**CXCR2 / IL-8 RB**	0,93	1,20	1,05	0,73	1,50
**gamma-Thrombin**	1,56	0,37	0,68	0,73	0,85
**Marapsin**	0,60	0,46	0,40	0,73	0,61
**CNTF**	0,88	1,04	0,94	0,73	1,23
**Cathepsin X/Z/P**	1,03	0,48	1,40	0,73	0,77
**CCR5**	1,54	2,67	0,57	0,73	0,62
**SART3**	1,10	1,09	0,89	0,73	0,88
**Kininostatin / kininogen**	0,48	0,52	0,70	0,73	0,46
**IL-20**	1,21	1,43	0,99	0,73	0,57
**PPARg2**	1,66	0,60	1,05	0,72	0,47
**67LR**	1,11	0,29	0,26	0,72	0,41
**IFN-gamma R1**	1,37	1,57	0,53	0,72	0,44
**MAP1A**	4,61	0,29	0,68	0,72	0,36
**CES1**	1,43	1,16	1,74	0,72	3,52
**PGAM2**	6,69	0,66	0,93	0,72	0,72
**TCCR / WSX-1**	0,61	1,25	0,50	0,72	0,46
**Nidogen-2**	0,88	0,90	0,87	0,72	0,80
**ZDHHC18**	0,26	0,18	0,46	0,72	0,15
**Utrophin**	0,91	1,07	1,01	0,72	0,83
**Chitobiase**	1,11	1,31	0,99	0,72	1,01
**Galanin**	0,65	0,68	1,06	0,72	0,91
**Thrombopoietin (TPO)**	0,80	0,86	1,25	0,72	1,24
**CD157**	0,46	0,98	0,45	0,72	0,76
**Quiescin Q6**	1,94	0,68	0,69	0,72	0,46
**PLC-gamma 1**	0,28	0,29	0,26	0,72	0,27
**TPP1**	2,48	0,79	1,60	0,72	1,32
**FGF-4**	0,94	1,62	0,38	0,72	0,19
**ESAM**	1,66	0,71	1,36	0,72	0,65
**Glutathione Synthetase/GSS**	1,21	0,35	0,41	0,72	0,38
**CPNE3**	1,10	1,22	1,31	0,72	1,44
**Somatotropin**	1,54	0,31	0,99	0,71	0,32
**PSA-Free**	0,83	1,10	1,16	0,71	1,29
**Procalcitonin**	1,20	0,54	1,19	0,71	1,18
**TWEAK / TNFSF12**	0,93	1,38	0,81	0,71	0,75
**FRY**	1,02	0,84	0,87	0,71	1,18
**CRTAC1**	0,90	0,75	0,77	0,71	1,21
**EV15L**	1,52	0,57	0,93	0,71	0,30
**Arp2**	0,75	0,42	0,51	0,71	0,46
**JARID2**	0,38	0,67	0,42	0,71	0,19

**PPOX**	0,49	1,55	1,00	0,71	0,43
**SERTAD2**	2,53	1,00	0,78	0,71	0,41
**C1s**	0,91	1,00	1,12	0,71	1,13
**Hemoglobin subunit beta/HBB**	3,82	0,69	0,16	0,71	0,92
**TMEFF2**	0,97	0,95	1,40	0,71	1,11
**PI 16**	0,63	0,49	0,60	0,71	0,36
**ADAMDEC1**	1,11	0,80	1,29	0,70	1,22
**BMPR-IA / ALK-3**	0,69	0,51	0,94	0,70	2,20
**Alpha 1 AG**	0,52	2,03	1,09	0,70	0,60
**MCMP2**	0,57	0,56	0,34	0,70	0,43
**DR6 / TNFRSF21**	0,44	0,64	0,29	0,70	0,72
**Complement factor H**	0,73	1,90	1,32	0,70	1,03
**Pappalysin-1**	0,67	3,34	0,68	0,70	0,50
**ErbB3**	1,60	2,41	1,10	0,70	1,29
**VCP**	1,27	0,94	0,97	0,70	1,17
**IGF-II**	0,67	0,72	1,23	0,70	0,69
**SSEA-1**	0,68	0,76	1,09	0,70	1,01
**GM-CSF R alpha**	1,65	2,37	0,66	0,70	0,48
**GPX1**	1,01	0,58	0,62	0,70	0,38
**PGM1**	5,76	0,37	0,46	0,70	0,57
**MCP-3**	3,71	4,25	0,48	0,70	0,78
**CRF21**	0,76	0,98	0,12	0,70	1,04
**CD74**	1,89	0,70	0,70	0,70	0,37
**Semenogelin I/SEMG1**	0,02	0,36	0,50	0,69	0,00
**Defensin**	4,48	0,07	0,41	0,69	0,46
**Ihh**	1,15	0,29	1,84	0,69	4,77
**ADAMTS-17**	1,43	1,11	0,91	0,69	0,32
**Serpin D1**	0,84	0,79	1,33	0,69	0,85
**Keratin 36**	2,76	0,65	0,88	0,69	0,55
**UNC45A**	2,71	1,14	0,91	0,69	0,86
**CFI**	0,96	0,57	1,16	0,69	0,69
**RPL22**	0,69	1,20	0,83	0,69	0,85
**COX-2**	0,55	1,14	0,40	0,69	0,76
**Contactin-1**	0,49	0,81	0,76	0,69	0,47
**KRT72**	1,24	0,42	0,42	0,69	0,29
**GPR-39**	0,56	0,57	1,09	0,69	0,59
**AMPKa1**	0,66	0,56	1,14	0,69	0,74
**MMP-9**	1,60	1,30	1,66	0,69	0,69
**Cystatin B**	1,11	0,66	0,88	0,69	0,86
**Annexin V**	0,62	0,32	0,35	0,69	0,75
**GCLC**	0,90	0,95	0,65	0,68	0,66
**TLR2**	0,67	4,82	0,27	0,68	0,86
**NCAM-1 / CD56**	0,58	0,66	0,71	0,68	0,30
**ADAMTS-1**	1,88	0,63	1,11	0,68	0,54
**Cystatin D**	0,81	0,79	1,07	0,68	0,86
**HPRT**	5,76	0,62	0,99	0,68	1,41
**Ornithine Carbamoyltransferase /OTC**	5,91	0,43	0,53	0,68	0,61

**IL-1 F6 / FIL1 epsilon**	0,71	1,12	1,39	0,68	0,85
**CCR2**	0,75	1,38	0,96	0,68	1,37
**Histone H2B K**	2,84	0,39	0,60	0,68	0,72
**Eotaxin-3 / CCL26**	1,63	2,20	0,56	0,68	0,60
**GLIPR2**	1,25	1,33	1,57	0,68	0,71
**IL-17F**	0,52	0,86	0,81	0,68	0,93
**BLVRB**	0,27	0,74	0,27	0,68	1,15
**PKLR**	8,44	0,38	0,56	0,68	0,43
**FGF R4**	0,90	1,26	1,11	0,68	1,27
**ROCK2**	0,91	0,56	0,52	0,68	3,51
**Calmodulin**	1,23	0,46	0,34	0,67	0,53
**TPX**	3,44	1,80	0,27	0,67	0,12
**EphA2**	0,98	0,80	1,32	0,67	0,95
**FBPase 1**	0,37	0,89	0,58	0,67	0,47
**Six3**	2,28	0,92	0,93	0,67	1,39
**ApoA2**	0,92	1,43	0,71	0,67	0,95
**Histone H2A**	4,06	0,65	0,72	0,67	0,78
**Fumarylacetoacetate hydrolase/FAH**	1,02	0,84	1,14	0,67	1,61
**ART3**	1,10	0,65	0,95	0,67	0,41
**HMGB3**	1,40	0,34	0,69	0,67	0,80
**hnRNP U**	3,99	0,47	0,70	0,67	1,07
**BIK**	0,23	0,52	0,25	0,67	0,53
**PLOD2**	0,78	0,54	1,00	0,67	1,29
**CK-MB**	1,55	1,83	0,78	0,67	0,27
**Ficolin-2**	0,86	0,55	1,02	0,66	0,98
**IL-23**	0,17	0,18	0,17	0,66	0,16
**MRP 1**	0,82	0,61	1,15	0,66	1,43
**Thioredoxin-1**	0,66	0,72	0,69	0,66	0,64
**PTPRS**	5,03	0,45	0,57	0,66	0,49
**Claudin-4**	1,01	0,92	1,26	0,66	0,98
**IGF-II R**	0,31	0,43	0,48	0,66	0,41
**C 1q S**	1,16	0,61	0,36	0,66	0,82
**Kallikrein 10**	0,81	0,25	0,51	0,66	0,26
**Aspartyl Aminopeptidase/DNPEP**	1,02	0,23	0,13	0,66	0,27
**EphA3**	1,23	0,50	1,14	0,66	0,56
**ENO1**	0,91	0,25	0,28	0,66	0,34
**IL-26**	1,19	0,57	1,93	0,66	3,50
**UBE2N/Ubc13**	0,65	0,67	0,59	0,66	0,72
**IL-5 R alpha**	0,85	1,61	0,73	0,66	0,48
**MMP-8**	0,85	0,70	0,92	0,66	1,42
**INSRR**	1,20	0,65	1,22	0,66	0,78
**IL-28A**	1,31	2,04	0,62	0,66	0,74
**CD16**	1,64	0,69	0,46	0,66	0,55
**CD36**	1,28	1,12	1,03	0,66	1,05
**Caspase-14**	1,38	0,67	1,85	0,66	3,65
**MYH7**	3,92	0,30	0,21	0,66	0,24
**IL-3 R alpha**	1,55	2,51	0,56	0,66	0,56

ADH4	0,86	0,29	0,51	0,65	0,30
SBSN	3,82	1,37	1,31	0,65	0,73
CL-P1	0,78	0,57	0,74	0,65	0,95
GCDFP 15	0,84	0,60	0,44	0,65	0,47
CrkL	1,12	1,17	0,74	0,65	2,16
Endoglin / CD105	0,72	1,14	0,31	0,65	0,43
ALBUMIN	0,82	1,02	0,84	0,65	0,88
CAP1	1,07	0,28	0,37	0,65	0,35
ApoH	1,78	0,73	1,13	0,65	0,65
Activin RIB / ALK-4	2,51	2,10	0,53	0,65	0,54
ApoC3	0,49	0,41	0,70	0,65	1,14
IBSP	0,40	0,57	0,40	0,65	0,30
p21	0,72	0,74	0,99	0,65	0,71
Chromogranin A	2,37	0,92	0,51	0,65	0,36
QPRT	0,29	0,79	0,54	0,65	0,31
KMD4B	3,06	0,52	0,80	0,64	0,57
PRELP	0,40	0,54	0,40	0,64	0,35
Fibulin 3	0,61	1,18	0,90	0,64	1,10
beta III Tubulin/CUBB3	0,78	0,30	0,59	0,64	0,36
GNPTG	1,33	0,70	1,22	0,64	1,22
PTEN	0,78	1,29	0,79	0,64	0,50
CD40 / TNFRSF5	1,16	0,99	1,18	0,64	1,10
Caspase-8	1,41	0,31	0,80	0,64	0,20
IDH3A	1,32	0,99	1,04	0,64	0,88
PSMA4	4,01	0,26	0,38	0,64	0,35
BAF57	1,08	0,80	0,78	0,64	1,10
Talin1	0,50	0,46	0,49	0,64	0,61
Glucosidase 2 subunit beta/PRKCSH	1,44	0,24	0,28	0,64	0,46
IL-27	0,27	0,35	0,29	0,64	0,23
Granzyme A	0,46	1,51	0,40	0,64	0,60
STAT3	0,45	0,33	0,51	0,64	0,31
Thrombin	0,89	0,67	0,96	0,63	0,97
CD38	0,66	0,79	1,25	0,63	0,96
VSIG4	0,55	1,09	0,79	0,63	1,18
Neuropilin-1	0,86	0,26	1,36	0,63	1,25
IL-17RC	0,40	0,84	0,80	0,63	0,78
Cathepsin H	0,41	0,49	0,38	0,63	0,58
Calbindin	0,88	0,76	1,09	0,63	1,11
Factor XIII A	0,94	1,18	1,15	0,63	1,23
FGF-17	0,98	0,50	0,44	0,63	0,35
CIP29	1,58	1,07	1,50	0,63	1,74
PI 3-Kinase C2 beta	0,42	0,21	0,34	0,63	0,27
Neurturin	0,66	0,53	1,07	0,63	0,65
MFRP	1,04	0,95	0,42	0,63	0,32
RHOC	1,18	0,67	0,60	0,63	0,50
IL-21 R	1,70	2,60	0,72	0,63	1,03
Histone H4	3,55	0,30	0,34	0,63	0,59

**Histone H3.3**	2,74	0,31	0,55	0,63	0,63
**IL-20 R beta**	0,40	0,64	0,47	0,63	0,38
**ADH5**	0,97	0,41	0,61	0,63	0,44
**Mer**	0,62	0,41	1,00	0,63	0,63
**Angiostatin**	1,03	1,22	1,16	0,63	1,23
**Prostasin**	0,79	0,73	1,21	0,63	1,13
**ADAS**	0,95	0,47	0,43	0,63	0,48
**Fascin**	0,34	0,32	0,37	0,63	0,63
**Apolipoprotein L 1**	0,66	0,24	0,38	0,63	0,21
**fast skeletal Myosin**	1,06	0,33	0,32	0,63	0,85
**APP**	0,50	0,77	0,68	0,63	0,53
**Pro-MMP-9**	1,45	0,67	1,11	0,63	0,43
**GASP-1 / WFIKKNRP**	0,69	0,66	1,07	0,62	0,70
**Olfactomedin-2**	0,68	0,42	0,38	0,62	0,56
**Artemin**	1,73	0,92	1,40	0,62	1,40
**PAI-1**	1,18	1,80	1,17	0,62	0,52
**Growth Hormone R (GHR)**	0,70	0,91	1,18	0,62	0,69
**Ctip2**	0,93	0,91	0,47	0,62	0,69
**Angiogenin**	1,14	1,42	0,90	0,62	0,41
**IL-1 R4 / ST2**	1,97	2,81	1,11	0,62	0,63
**Grb2**	0,99	6,69	0,56	0,62	1,21
**LBP**	0,55	0,69	0,73	0,62	0,43
**SORD**	0,81	1,61	0,71	0,62	1,09
**Decorin**	0,77	1,40	0,43	0,62	0,26
**IL-17R**	1,50	2,18	0,68	0,62	0,72
**LH**	1,45	1,73	0,38	0,62	0,15
**Thrombospondin-1**	0,98	0,79	1,44	0,62	0,93
**Serotonin**	0,84	0,89	0,29	0,62	0,49
**Kilon**	0,35	0,44	0,26	0,62	0,47
**Acetyl-CoA acetyltransferase/ACAA**	0,83	0,43	0,54	0,62	0,55
**Cathepsin S**	0,45	1,01	0,43	0,62	0,33
**KCC3**	0,84	1,09	0,64	0,62	0,55
**C1RL**	0,88	1,30	1,20	0,61	1,30
**PF4 / CXCL4**	1,12	1,54	1,34	0,61	0,65
**ASH2L**	0,91	0,63	0,67	0,61	0,67
**Fractalkine**	1,62	1,95	0,58	0,61	0,37
**Positive Control**	0,91	0,91	1,15	0,61	0,92
**PDE1B**	0,69	1,54	1,07	0,61	0,59
**Positive Control**	0,92	0,90	1,19	0,61	0,97
**Cathepsin A**	1,03	0,52	0,79	0,61	1,24
**Follistatin**	0,81	0,97	1,25	0,61	0,77
**CCT3**	0,83	0,69	0,72	0,61	0,57
**C4BPA**	1,00	0,42	0,35	0,61	0,33
**MFAP4**	0,48	2,03	0,56	0,61	0,22
**Positive Control**	0,91	0,89	1,16	0,61	0,89
**UROC1**	2,00	0,70	0,89	0,61	1,51
**UNC5H4**	0,80	0,76	0,65	0,61	0,75

**SAA**	0,55	0,25	1,03	0,61	0,46
**IGFBP-4**	1,30	2,00	0,49	0,61	0,51
**SNCG**	1,41	3,16	0,80	0,61	2,36
**HTRA2**	1,01	1,23	0,81	0,61	1,01
**Cytokeratin 19**	1,27	0,54	0,82	0,61	0,61
**FADD**	0,70	0,77	0,45	0,60	0,55
**FBP2**	1,03	0,25	0,52	0,60	0,40
**HIP1R**	0,88	0,94	1,90	0,60	1,30
**Fibronectin**	0,72	1,06	1,42	0,60	0,95
**Positive Control**	0,88	0,90	1,15	0,60	0,92
**ATP5A**	0,73	0,32	0,41	0,60	0,34
**DPPIV**	0,63	1,02	0,95	0,60	1,04
**Clusterin**	0,79	1,01	0,96	0,60	2,12
**Frizzled-4**	0,53	1,76	0,27	0,60	0,74
**CXCL16**	0,42	0,69	0,50	0,60	0,60
**MMP-16 / MT3-MMP**	0,22	0,56	0,30	0,60	0,40
**Osteocrin**	0,19	0,13	0,17	0,60	0,11
**Filamin C**	0,94	0,22	0,31	0,60	0,28
**Desmin**	0,93	1,17	1,35	0,60	2,11
**CRMP2**	0,91	0,27	0,37	0,60	0,31
**RPLP0**	1,53	0,58	0,56	0,60	0,52
**CD30 Ligand / TNFSF8**	0,42	0,27	0,66	0,60	0,49
**AKR7A2**	0,80	0,28	0,72	0,59	0,53
**CD24**	0,76	0,63	0,98	0,59	0,90
**sFRP-4**	0,31	1,25	0,30	0,59	0,33
**Activin RIA / ALK-2**	2,14	1,71	0,68	0,59	0,64
**BAFF**	1,40	0,00	0,57	0,59	0,00
**PA2G4**	0,46	0,27	0,38	0,59	0,39
**EHD3**	0,62	0,36	0,78	0,59	0,43
**IFN-alpha / beta R2**	0,21	0,39	0,19	0,59	0,23
**TSG-6**	1,58	1,46	3,57	0,59	2,68
**PGRP-S**	0,60	0,69	0,94	0,59	0,49
**Plasminogen**	0,70	0,96	1,29	0,59	1,07
**Neurogranin**	0,55	0,25	1,45	0,59	1,04
**FGF-8**	2,27	0,52	0,44	0,59	0,15
**NT5C3**	4,48	0,36	0,38	0,59	0,37
**Fc RIIB/C**	0,71	0,51	1,19	0,59	0,77
**LASP1**	0,47	0,24	0,18	0,59	0,72
**Ezrin**	0,31	0,71	0,20	0,59	0,50
**PDGF-C**	0,23	0,22	0,17	0,59	0,14
**PRTN3**	0,93	0,64	1,11	0,59	0,81
**HSP32**	0,79	0,80	1,45	0,59	0,79
**IL-22 R**	1,75	2,61	0,77	0,59	0,68
**ANGPTL4**	0,43	0,56	0,30	0,59	0,19
**CCDC126**	1,11	0,87	1,33	0,59	1,63
**ATPB**	0,75	0,54	0,64	0,58	0,81
**MBL**	0,70	0,91	1,19	0,58	0,74

**ALAD**	0,66	0,20	0,34	0,58	0,20
**TOP2B**	1,91	0,78	1,03	0,58	0,85
**HLA-C**	0,49	0,98	0,99	0,58	0,92
**PCSK9**	2,08	0,95	0,91	0,58	0,72
**Calumenin**	0,97	0,33	0,32	0,58	0,32
**Netrin G2**	0,47	0,31	0,53	0,58	0,37
**DDAH1**	0,43	0,33	0,44	0,58	0,54
**PIN**	0,57	0,28	0,56	0,58	0,25
**ARPC2**	0,83	0,38	0,45	0,58	0,41
**TUBA6**	0,79	1,14	0,75	0,58	0,75
**IL-17B**	0,78	0,41	0,94	0,58	0,51
**D6**	0,71	3,20	0,85	0,58	0,59
**Glut5**	0,76	0,38	1,10	0,57	0,40
**CFHR5**	1,24	1,71	1,18	0,57	1,10
**PDIA6**	0,71	1,89	1,13	0,57	0,46
**Mammaglobin A**	2,08	0,65	0,43	0,57	0,35
**Factor XIII**	0,48	0,29	0,45	0,57	1,87
**C 1q**	0,71	0,76	0,68	0,57	0,83
**IL-36RN**	1,20	0,47	1,06	0,57	0,71
**URB**	0,65	0,97	0,46	0,57	0,88
**TSLP R**	0,83	1,14	0,55	0,57	0,80
**Frizzled-6**	0,35	0,76	0,28	0,57	0,45
**IL-1 F9 / IL-1 H1**	0,58	1,81	0,67	0,57	0,51
**BDNF**	0,56	1,05	0,99	0,57	1,20
**CECR1**	3,50	0,00	2,16	0,57	6,67
**MIP-3 beta**	0,41	0,73	0,38	0,57	0,56
**TAB182**	0,49	0,77	0,74	0,57	0,73
**Neurotrimin**	1,60	0,78	0,79	0,57	0,67
**DBI**	0,66	0,73	0,88	0,57	0,50
**alpha Tubulin**	0,67	0,42	0,99	0,57	0,33
**Lamin B1**	7,08	0,30	0,28	0,57	0,20
**TRP-1**	0,44	0,57	0,38	0,57	0,57
**GATA-4**	0,65	0,61	0,70	0,57	0,83
**Collagen VI**	0,53	0,66	0,51	0,57	0,45
**B3GNT1**	0,65	0,50	0,94	0,57	0,75
**ArgRS**	0,73	0,39	0,39	0,56	0,45
**Histone H1.3**	5,89	1,17	0,58	0,56	0,90
**GST**	2,12	0,34	0,91	0,56	0,43
**Desmocollin-2**	0,40	0,31	0,25	0,56	0,41
**TRA-1-60**	0,97	0,86	1,51	0,56	0,69
**ATP5O**	0,67	0,77	0,56	0,56	1,34
**Arp3**	0,80	0,32	0,42	0,56	0,31
**LPS**	1,22	0,53	0,71	0,56	1,32
**ACTC1**	0,69	1,34	0,59	0,56	0,83
**Antithrombin III**	0,84	1,00	1,18	0,56	1,09
**ABI3BP**	1,04	0,53	1,36	0,55	2,25
**Band 3**	1,07	0,26	0,42	0,55	0,31

AFP	0,91	0,88	0,75	0,55	0,40
C3orf75	0,77	0,35	1,16	0,55	1,29
mTOR	0,42	1,01	0,89	0,55	0,49
CCR1	0,69	0,67	0,73	0,55	0,98
DCBLD2	0,32	0,66	0,32	0,55	0,23
Cytokeratin 5	1,21	0,36	0,46	0,55	0,43
Progranulin	0,50	0,95	0,82	0,55	0,45
HIBADH	0,45	0,46	1,60	0,55	1,02
LDHA	0,27	0,63	0,24	0,55	0,30
SDF-1 / CXCL12	1,69	1,73	0,77	0,55	1,04
DSCAM	0,46	0,73	0,71	0,55	1,27
IL-1 R8	0,25	0,29	0,23	0,55	0,44
ErbB4	0,89	0,76	1,00	0,55	0,60
Trappin-2	0,26	0,66	0,68	0,54	0,26
UROD	0,25	0,44	0,41	0,54	1,23
Corneodesmosin	0,30	0,44	0,24	0,54	0,41
ApoA1	0,49	0,55	0,53	0,54	0,53
S100A8	0,33	0,56	0,36	0,54	0,27
SIRP beta 1/CD172b	0,56	0,56	0,30	0,54	0,35
IQGAP1	1,70	0,25	0,44	0,54	0,24
OBCAM	0,69	1,12	0,77	0,54	0,83
Cezanne	0,75	0,75	0,96	0,54	0,69
ARFGEF3	0,36	0,29	0,82	0,54	0,33
MMP-13	1,52	1,88	0,24	0,54	0,13
GFR alpha-3	0,71	1,46	0,94	0,54	0,50
CLIC1	0,73	0,50	0,97	0,54	0,55
Spectrin beta-5	0,62	1,26	0,63	0,54	0,71
DISC 1	0,40	0,37	0,41	0,54	0,52
GSTP1	1,39	0,77	1,56	0,54	2,43
Contactin-3	1,60	0,50	1,99	0,54	2,47
Activin C	0,64	0,65	0,92	0,54	0,83
BMP-9	0,78	0,51	0,81	0,54	0,86
IL-17RD	0,35	0,29	0,45	0,54	0,30
FTL	0,69	0,55	0,64	0,54	1,03
CD21	1,24	1,87	0,95	0,54	1,17
D-Dimer	1,44	0,38	0,89	0,54	0,38
CLEC3B	0,74	0,91	0,94	0,54	0,76
glutathione S transferase Omega 1/GSTO1	1,15	0,57	0,58	0,54	0,94
CD40 Ligand / TNFSF5 /CD154	0,75	0,74	0,75	0,54	1,20
ApoM	0,88	0,70	0,46	0,53	0,36
ALS+B62:B1212CR1	0,88	0,36	0,55	0,53	0,25
LAIR1	0,26	0,72	0,36	0,53	1,16
CD46	2,65	0,58	0,44	0,53	0,31
SLPI	1,94	0,48	0,37	0,53	0,28
MPO	0,83	0,60	0,84	0,53	0,55
ALDH16A1	0,75	0,19	0,30	0,53	0,21
CART	0,43	0,49	0,72	0,53	0,40

HE4	0,54	0,36	1,03	0,53	0,43
p39	0,39	0,92	0,64	0,53	0,36
CFVII	0,67	1,72	0,81	0,53	2,27
Vitronectin	0,75	0,50	0,85	0,53	0,52
CD133	1,67	0,85	1,27	0,53	1,78
Fyn	1,03	0,19	0,72	0,53	0,09
KLK-B1	0,56	0,54	0,66	0,53	1,22
FUCA1	0,92	1,07	1,23	0,53	2,21
Cytokeratin 20	0,82	0,39	0,46	0,53	0,45
Chemerin	0,62	0,45	0,95	0,53	0,51
Als2	0,70	0,31	0,36	0,53	0,33
Haptoglobin	0,65	1,06	1,51	0,53	0,76
PPCS	6,51	0,57	0,84	0,53	0,37
Caldesmon/CALD1	0,78	0,78	0,90	0,53	0,83
Hemoglobin subunit delta/HBD	0,40	0,41	0,95	0,53	0,77
GDF-15	0,47	0,72	0,88	0,53	0,80
EEF1G	1,04	0,11	0,39	0,53	0,28
ENSA	0,85	0,18	0,40	0,53	0,39
Cytokeratin 3	1,34	0,10	1,16	0,53	0,64
APA	0,68	0,29	0,47	0,52	0,13
Alcohol Dehydrogenase/ADH	0,87	0,29	0,13	0,52	0,31
PDGF R beta	1,45	2,33	0,46	0,52	0,19
ACAA1	0,75	0,58	0,73	0,52	0,49
FGFR1 alpha	0,77	0,29	0,86	0,52	0,48
PER1	0,33	0,25	0,27	0,52	0,31
Tcf20	1,71	0,58	0,61	0,52	0,38
IL-1 R9	0,35	0,29	0,29	0,52	0,38
Cytochrome c	0,61	0,90	0,73	0,52	1,43
EN-RAGE	0,31	0,46	0,86	0,52	0,40
ITIH4 a	0,59	0,56	0,81	0,52	0,69
HRSP12	1,19	0,56	1,53	0,52	1,73
AK2	0,84	0,14	0,33	0,51	0,29
CD9	1,01	0,32	0,59	0,51	0,40
GOT2	0,86	0,40	0,35	0,51	0,89
NQO2	0,40	0,78	0,75	0,51	0,51
GMNN	1,62	0,85	0,80	0,51	0,80
PSMA1	0,87	0,73	1,23	0,51	0,66
ALKP	0,92	0,58	0,86	0,51	0,57
Eotaxin-2 / MPIF-2	1,24	2,23	0,57	0,51	0,58
Endocan	0,34	1,23	0,32	0,51	0,29
KLF4	0,39	0,96	0,31	0,51	0,44
Layilin	0,53	0,48	0,72	0,51	0,79
COTL1	0,68	0,45	0,95	0,51	0,60
CBS	0,70	0,31	0,45	0,51	0,34
COL19A1	0,72	0,65	1,00	0,51	0,85
MFG-E8	0,66	0,45	0,96	0,51	0,54
Semenogelin II/SEMG2	2,23	0,60	0,86	0,51	0,48

**Syntaxin 7**	0,42	1,07	0,45	0,51	0,45
**Lipocalin-2**	0,87	0,60	1,57	0,51	0,67
**BCMA / TNFRSF17**	0,61	0,61	0,52	0,51	0,59
**IL-1 F10 / IL-1HY2**	0,30	0,53	0,20	0,51	0,38
**SH3BGRL**	1,84	0,89	0,37	0,51	0,70
**S100A6**	0,33	0,52	0,52	0,51	0,37
**Talin1&2**	0,35	0,68	0,69	0,51	0,43
**Siglec-1**	0,58	0,61	0,44	0,51	0,51
**PIK3IP1**	0,82	0,46	0,74	0,51	0,76
**Endorphin Beta**	1,48	1,65	0,54	0,50	0,35
**C8G**	0,64	0,63	0,68	0,50	0,34
**KIF5B**	3,35	0,33	0,31	0,50	0,36
**cTnT**	1,39	1,79	0,53	0,50	0,28
**GNB1**	1,23	0,09	0,43	0,50	0,51
**C3a**	0,62	1,99	1,03	0,50	5,20
**CA1**	0,62	0,50	0,86	0,50	0,77
**pIgR**	1,68	0,35	0,58	0,50	0,39
**Glypican 3**	0,32	0,53	0,26	0,50	0,29
**MEP1A**	0,69	0,41	0,76	0,50	0,99
**alpha Glucosidase II**	0,82	0,06	0,38	0,50	0,42
**CapG**	0,41	0,76	0,31	0,50	0,64
**FGF-11**	0,83	0,86	1,36	0,50	0,83
**Frizzled 8**	0,62	0,14	0,25	0,50	0,22
**ALPP**	0,70	0,59	0,87	0,50	1,77
**Ceruloplasmin**	0,62	0,41	0,60	0,50	0,43
**GOLPH4**	0,96	0,22	0,19	0,50	0,29
**Galectin-3**	0,59	0,86	0,77	0,50	0,59
**Vitamin K-dependent protein S**	0,51	1,13	0,51	0,50	0,39
**IL-12 R beta 2**	0,16	0,22	0,22	0,49	0,40
**CHORDC1**	0,84	0,70	0,77	0,49	0,69
**Hemoglobin**	1,22	0,85	0,76	0,49	1,50
**CCR4**	0,60	0,59	0,82	0,49	0,82
**PLUNC**	0,67	0,61	1,10	0,49	0,90
**ATBF1/ZFHX3**	0,46	0,58	0,45	0,49	0,90
**CEA**	0,98	1,24	0,55	0,49	0,36
**NRG1 Isoform GGF2**	0,37	0,81	0,27	0,49	0,38
**CFHR4**	0,74	0,83	1,01	0,49	0,97
**Glut1**	0,56	0,59	0,96	0,49	0,68
**EHD1**	0,68	0,14	0,34	0,49	0,31
**CXCR5 /BLR-1**	0,81	0,49	0,75	0,49	0,60
**COPS8**	0,92	0,28	0,74	0,49	0,51
**C1QB**	0,68	0,25	0,24	0,49	0,24
**Collagen IX**	0,74	0,43	1,16	0,49	0,45
**IL-1 R6 / IL-1 Rrp2**	0,64	0,96	0,85	0,49	0,94
**Calsyntenin-1**	0,40	0,44	0,36	0,49	0,27
**TL1A / TNFSF15**	0,70	1,29	1,11	0,49	1,80
**HSP27**	1,34	0,26	0,64	0,49	0,22

**PCCA**	0,76	0,43	0,98	0,49	1,64
**GFR alpha-4**	0,83	1,17	1,00	0,49	0,69
**MYH2**	5,55	0,73	0,21	0,49	0,27
**Plexin B2**	1,01	0,48	0,92	0,49	0,70
**ACK1**	0,63	0,29	0,97	0,49	0,32
**Fetuin A**	0,71	0,56	0,66	0,49	0,67
**FGF Basic**	0,57	0,53	1,02	0,48	0,73
**PDGF-AB**	1,77	1,15	0,45	0,48	0,62
**ELAVL1**	1,40	0,69	0,51	0,48	0,54
**Apolipoprotein F**	0,66	0,50	0,48	0,48	0,37
**Integrin alpha V**	0,65	0,11	0,77	0,48	0,55
**BMPR-II**	0,65	0,53	0,83	0,48	0,87
**Mcl-1**	0,53	0,53	0,81	0,48	0,96
**FGF-13 1B**	0,88	0,58	0,89	0,48	0,85
**ASXL1**	0,94	0,62	0,85	0,48	1,31
**IGFBP-3**	0,85	0,68	0,82	0,48	0,67
**HMGN2**	0,53	0,62	0,74	0,48	0,80
**Cytokeratin 1**	1,49	0,85	0,86	0,48	0,70
**FGF-19**	0,40	0,91	0,24	0,48	0,46
**CCR9**	0,92	0,91	0,74	0,48	0,93
**Cyclophilin A**	0,81	0,58	0,90	0,48	1,33
**IL-17B R**	0,83	0,64	0,93	0,48	0,80
**Aspartate Aminotransferase /AST**	0,57	0,39	0,46	0,48	0,23
**PPP2R5C**	0,86	1,11	0,71	0,48	1,22
**LRG1**	0,73	0,74	0,73	0,47	0,54
**Frizzled-5**	0,19	0,22	0,25	0,47	0,33
**PTHLP**	2,18	2,35	0,47	0,47	2,87
**CD45**	1,34	1,30	0,40	0,47	0,25
**SPARC**	0,58	0,80	0,67	0,47	0,58
**BIN2**	0,66	0,79	0,96	0,47	1,29
**TAX1BP3**	0,48	0,85	0,58	0,47	0,61
**APC**	1,50	1,05	0,38	0,47	0,18
**Collagen V**	0,56	0,68	0,81	0,47	0,41
**Calpastatin**	1,20	0,74	1,95	0,47	2,45
**DDT**	1,03	1,02	0,95	0,47	2,38
**Flt-3 Ligand**	0,76	1,21	0,33	0,47	0,13
**TGF-beta 1**	1,55	1,52	0,72	0,47	0,41
**BMP-5**	0,91	0,74	0,67	0,47	0,72
**ALDH9A1**	1,32	0,54	1,23	0,47	0,51
**ECHS1**	0,61	0,24	0,28	0,47	0,29
**CHI3L1**	1,10	0,22	0,76	0,47	0,31
**Nesprin2**	0,52	0,79	0,58	0,47	0,43
**GASP-2 / WFIKKN**	0,50	0,69	0,84	0,47	0,50
**MARCKS**	0,63	0,48	0,78	0,47	0,54
**FGF-12**	0,71	0,44	0,39	0,47	0,39
**KSR1**	1,61	0,14	0,31	0,47	0,17
**L-Selectin (CD62L)**	0,93	0,79	0,51	0,47	0,23

Activin RII A/B	1,14	0,59	0,79	0,46	1,09
Prouroguanylin	1,00	0,41	0,92	0,46	1,33
EG-VEGF / PK1	1,09	1,09	1,46	0,46	0,84
KRT82	0,61	0,47	0,94	0,46	1,12
Annexin A7	0,45	0,54	0,27	0,46	0,26
CA 125	0,92	0,74	0,75	0,46	0,28
C-peptide	0,78	0,57	0,70	0,46	0,44
BMP-8	0,89	0,68	1,08	0,46	1,35
GRO	0,85	1,14	0,76	0,46	0,58
Adiponectin / Acrp30	0,81	0,98	1,18	0,46	0,81
CXCR6	0,91	0,49	0,94	0,46	0,61
A2M	1,09	2,03	0,43	0,46	1,25
GLP-1	1,48	0,35	0,84	0,46	0,32
Thymosin b10	0,41	0,56	0,39	0,46	0,40
PABP	1,30	0,58	0,86	0,46	0,56
HTRA1	0,37	0,32	0,80	0,46	0,71
CLTA	0,71	0,81	1,07	0,46	1,47
Transthyretin	0,41	0,86	0,42	0,46	0,58
CD71	1,29	0,45	1,35	0,46	0,20
Cytokeratin 13	1,00	0,52	0,69	0,46	0,52
Troponin C	1,19	1,67	0,40	0,46	0,09
Hoxb3	1,03	0,63	0,30	0,46	2,67
MAGP-2	0,24	0,44	0,35	0,46	0,28
RPS10	0,17	0,61	0,42	0,46	0,18
PCMT1	0,52	0,62	0,63	0,46	0,50
LAF4	0,99	0,41	0,56	0,46	0,39
Cytokeratin 4	0,64	0,57	0,61	0,46	0,51
Fas Ligand	0,76	2,01	0,70	0,45	0,78
ADAMTS-15	1,12	0,43	0,89	0,45	0,35
Spinesin	0,13	0,09	0,15	0,45	0,09
Prdx6	0,53	0,25	0,26	0,45	0,11
Mimecan	0,40	0,43	0,24	0,45	0,42
APN	0,83	0,38	0,35	0,45	0,30
FSH	0,96	0,70	0,94	0,45	1,58
FGF-21	0,24	0,58	0,24	0,45	0,46
TXK	0,46	1,54	0,83	0,45	0,73
BRD2	0,95	0,22	0,28	0,45	0,42
Insulysin / IDE	0,37	0,53	0,28	0,45	0,28
IL-1 R3 / IL-1 R AcP	0,58	0,57	0,73	0,45	0,49
C5/C5a	0,73	2,52	0,74	0,45	2,26
Creatinine	0,36	0,69	0,46	0,45	0,24
p73	3,83	0,45	0,35	0,45	0,32
sFRP-1	0,32	0,84	0,50	0,45	0,48
TGM3	0,37	0,79	0,37	0,45	0,40
Fibrinopeptide A	0,89	0,46	0,87	0,45	0,40
STOM	0,30	0,89	0,37	0,45	0,33
Human Agrin	0,87	0,39	0,68	0,45	0,30

Caspase-3	0,24	2,73	0,53	0,44	0,27
Multimerin 2	0,65	0,52	1,02	0,44	1,23
Nectin-1	0,54	0,40	0,65	0,44	0,74
PF4V1	0,79	0,68	0,78	0,44	0,88
CD109	0,31	0,74	1,14	0,44	1,41
RPS28	1,07	0,47	0,61	0,44	0,47
Myosin 18B	2,08	0,60	0,61	0,44	0,37
Angiopoietin-1	0,64	0,36	0,87	0,44	0,47
CCR8	0,86	0,95	0,81	0,44	1,07
CAD	0,51	0,72	0,94	0,44	0,35
GDA	0,88	0,88	1,24	0,44	1,71
GFR alpha-2	0,76	0,68	1,04	0,44	0,96
IL-12 R beta 1	0,68	0,99	0,67	0,44	0,59
PRSS3	1,00	0,49	0,86	0,44	1,16
Hepassocin	0,64	0,95	0,84	0,44	0,40
DARS2	0,49	0,42	0,58	0,44	0,44
PSMD1	1,08	0,54	0,42	0,44	0,30
CA 19-9	0,93	0,41	0,75	0,44	0,24
IL-4 R	0,67	0,44	0,82	0,43	0,65
CS	0,78	0,36	0,45	0,43	0,44
Dkk-3	1,03	1,39	0,76	0,43	0,70
alpha Actinin 4	0,73	0,33	0,48	0,43	0,27
AHNAK	1,01	0,79	1,20	0,43	0,71
CRP	0,44	0,78	0,67	0,43	0,46
Intergrin a6	0,29	0,50	0,18	0,43	0,23
Ephrin B1	0,33	0,39	0,51	0,43	0,37
LEDGF	0,41	0,28	0,35	0,43	0,45
Selenoprotein P	0,51	1,90	1,04	0,43	1,74
ANGPTL3	0,71	0,27	0,92	0,43	0,64
ESD	0,95	0,13	0,43	0,43	0,26
Carboxypeptidase B2/CPB2	0,77	0,29	0,27	0,43	0,26
F11	0,77	0,60	1,00	0,43	0,51
ANGPTL6	1,10	1,11	1,44	0,43	1,18
MyBPC3	0,40	0,75	0,41	0,43	0,57
IQGAP2	1,26	0,27	0,57	0,43	0,32
TLR3	0,44	1,36	0,38	0,43	0,37
CALML5	0,68	0,35	0,73	0,42	0,46
Kallikrein 2	1,14	0,20	0,68	0,42	0,31
Cripto-1	0,83	0,96	0,65	0,42	0,53
HCR / CRAM-A/B	0,89	0,65	0,82	0,42	0,60
Mannosidase II	0,63	0,45	0,81	0,42	0,72
Catalase	0,50	0,75	0,27	0,42	0,96
Neurokinin-A	0,38	0,32	0,97	0,42	0,40
ApoA4	0,60	0,46	1,13	0,42	0,64
IL-13 R alpha 1	1,16	0,70	0,76	0,42	0,47
HRG-alpha	0,27	0,27	0,25	0,42	0,38
Advillin-N-t	0,69	0,49	1,92	0,42	1,52

**IL-1 sRII**	1,06	0,63	0,43	0,42	0,35
**BLAME**	0,46	0,55	0,80	0,42	0,73
**KIAA0319L**	0,80	0,59	1,05	0,42	0,57
**RREB1**	0,42	1,35	0,38	0,41	0,89
**TSR2**	0,49	0,91	0,67	0,41	0,41
**AgRP**	1,56	1,32	0,32	0,41	0,63
**MAPRE1**	0,71	0,39	0,76	0,41	0,37
**Keratin 38**	0,88	0,34	0,48	0,41	1,53
**LAM b1**	0,41	1,16	1,00	0,41	0,57
**Aldolase A**	0,44	0,52	0,87	0,41	0,26
**LAP3**	1,25	0,11	0,23	0,41	0,05
**FLG2**	0,64	0,41	0,44	0,41	0,55
**CutA**	1,60	0,55	0,69	0,41	0,33
**Chordin-Like 2**	0,95	0,80	1,26	0,41	1,33
**Fibrillin 1**	1,11	0,25	0,45	0,41	0,71
**FGF-9**	0,72	0,46	1,15	0,41	0,47
**FGF-6**	0,55	0,66	0,46	0,41	0,31
**ISOC2**	1,29	0,44	0,53	0,41	0,46
**GOLPH2**	0,68	0,23	0,29	0,40	0,29
**Protein p65**	0,53	0,78	0,65	0,40	0,32
**KHSRP**	1,71	0,11	0,29	0,40	0,10
**SPARCL1**	0,54	0,45	0,67	0,40	0,42
**RPS5**	0,88	0,67	0,63	0,40	0,45
**BAFF R / TNFRSF13C**	0,42	0,42	0,22	0,40	0,49
**Cytochrome b5**	0,65	0,22	0,21	0,40	0,23
**IGFBP-5**	1,47	1,95	0,27	0,40	0,16
**POR**	0,77	0,75	1,26	0,40	0,82
**HBZ**	0,95	0,52	0,96	0,40	1,38
**CXCL14 / BRAK**	0,66	0,75	0,78	0,40	1,31
**CXCR4 (fusin)**	0,97	0,62	0,86	0,40	0,77
**BPGM**	0,81	0,46	0,67	0,40	0,27
**Proteasome beta 1**	0,41	0,43	0,31	0,40	0,40
**IL-15 R alpha**	0,52	0,52	0,71	0,40	0,55
**G6PD**	0,51	0,30	0,77	0,40	0,45
**BMPR-IB / ALK-6**	0,74	0,87	0,70	0,39	0,98
**BMP-2**	0,68	0,40	0,49	0,39	0,64
**Myotrophin**	0,76	0,40	0,62	0,39	0,49
**LAD**	1,20	0,03	0,04	0,39	0,24
**MUCDHL**	0,33	0,21	0,37	0,39	0,30
**Profilin 1**	0,99	0,30	0,64	0,39	0,46
**COG4**	0,57	0,19	0,29	0,39	0,22
**IL-20 R alpha**	1,21	1,64	1,25	0,39	0,88
**HGFA**	0,72	0,61	0,83	0,39	1,38
**ADAMTS-18**	0,96	0,41	0,82	0,39	0,33
**LILRA3**	0,31	0,35	0,54	0,39	0,64
**Angiopoietin-like Factor**	1,37	0,38	0,77	0,39	0,96
**Nectin-3**	0,66	0,54	0,77	0,38	0,55

**Chordin-Like 1**	0,68	0,92	0,78	0,38	1,36
**BPIL1**	0,37	0,93	0,65	0,38	0,61
**PSMB5**	1,03	0,35	0,34	0,38	0,21
**LOK**	1,01	0,56	0,30	0,38	0,22
**ZC3H8**	0,57	0,44	0,46	0,38	0,46
**TNF-beta**	0,83	0,76	0,45	0,38	0,41
**CD98**	0,64	0,46	0,64	0,38	0,82
**PTP gamma**	0,56	0,68	0,68	0,38	0,71
**IL-11**	0,79	0,29	0,51	0,38	0,23
**Latent TGF beta bp2**	0,32	0,36	0,23	0,38	0,32
**CCR6**	0,52	0,45	0,88	0,38	0,68
**CPM**	0,87	0,71	1,02	0,38	0,78
**LUZP1**	0,59	0,53	0,89	0,38	1,22
**Cathelicidin**	0,88	0,91	0,52	0,38	0,70
**MDH2**	0,48	0,20	0,09	0,38	0,17
**MCAM**	0,88	1,21	0,46	0,38	0,97
**CBP**	1,10	0,68	0,64	0,38	0,35
**ARPC3**	0,54	0,49	0,87	0,38	0,63
**S-100b**	1,21	1,13	0,39	0,37	0,12
**GDF1**	0,81	0,75	0,98	0,37	0,86
**OIT3**	0,49	0,32	0,71	0,37	0,48
**FKBP51**	0,81	0,45	1,02	0,37	0,92
**CD48**	0,20	0,44	0,27	0,37	0,92
**PISD**	0,68	0,45	0,68	0,37	0,47
**BMP-3**	0,56	0,44	0,63	0,37	0,65
**Follistatin-like 1**	0,79	1,28	1,30	0,37	0,81
**C6 -N-t**	0,82	0,60	1,27	0,37	1,06
**SHMT1**	0,14	0,58	0,53	0,37	0,30
**GATM - C-terminal**	0,65	0,37	0,49	0,37	0,47
**GADD45A**	1,16	0,82	0,30	0,37	0,08
**GRHPR**	0,42	0,80	0,53	0,37	0,46
**Aconitase 1**	0,49	0,88	0,87	0,37	0,88
**ErbB2**	0,94	0,35	0,80	0,36	0,35
**ABL1**	0,68	0,46	0,75	0,36	0,66
**PCDX8**	0,55	0,35	0,87	0,36	0,63
**Btk**	0,51	0,27	0,82	0,36	0,31
**CA 15-3**	1,02	1,49	0,34	0,36	0,19
**HPR**	1,90	0,39	0,72	0,36	1,16
**STI1**	0,59	0,26	0,29	0,36	0,31
**SBP-1**	0,37	0,70	0,59	0,36	0,28
**CCL28 / VIC**	0,81	1,48	0,81	0,36	0,22
**EDAR**	0,52	0,56	0,43	0,36	0,51
**FAM3B**	0,48	0,38	0,51	0,36	0,38
**B7-H2**	0,54	0,64	0,75	0,36	0,75
**EVC2**	0,59	0,53	0,62	0,36	0,19
**Endothelin Receptor A**	0,44	0,31	0,86	0,36	0,17
**HDGF**	0,97	1,03	0,54	0,36	1,26

MINA	0,42	1,24	0,83	0,36	0,65
ARP2/3	0,51	0,42	0,31	0,35	0,50
RET	0,67	0,42	0,49	0,35	0,17
CLIC4	0,53	0,20	0,59	0,35	0,25
Cytokeratin 16	0,61	0,34	0,58	0,35	0,49
CHREBP	0,39	0,89	0,90	0,35	0,45
FGF-5	0,87	1,27	0,88	0,35	0,55
ITGB4BP	0,20	0,49	0,64	0,35	0,14
MBD2	2,24	0,16	0,35	0,35	0,18
ENO1 + ENO2 + ENO3	0,52	0,32	0,51	0,35	0,34
CD59	0,99	0,52	0,81	0,35	0,43
Erythropoietin	1,02	0,67	0,98	0,35	0,65
Proteasome 20S b7	0,94	0,32	0,56	0,35	0,29
C4.4A	0,37	1,02	0,31	0,35	0,81
EGF R / ErbB1	1,41	1,10	0,43	0,35	0,57
SMA	0,23	0,61	0,43	0,35	0,36
MINPP1	0,17	1,18	0,19	0,34	0,12
APLP-1	0,83	0,51	1,47	0,34	1,51
Col6A2	0,67	0,27	0,35	0,34	0,23
PLOD1	0,76	0,18	0,22	0,34	0,44
GDF7	0,67	0,21	0,36	0,34	0,36
IL-23p19	0,68	0,22	0,49	0,34	0,15
ERp72	0,44	0,31	0,36	0,34	0,16
Angiopoietin-like 1	0,72	0,69	0,92	0,34	0,70
LTA4H	0,26	0,55	0,20	0,34	0,25
MMP-10	1,07	0,35	0,92	0,34	0,36
MATK	0,29	1,31	0,53	0,34	0,18
IL-23 R	0,33	0,70	1,23	0,34	0,56
RSU1	0,18	0,63	0,49	0,34	0,30
MASP3	0,43	0,32	0,72	0,34	0,58
EDG-1	0,98	0,62	1,39	0,34	0,76
CENPF	0,53	0,55	0,32	0,34	0,48
Coagulation Factor III / Tissue Factor	0,63	0,52	0,62	0,34	0,55
BMP-15	0,50	0,52	0,41	0,34	0,59
Filamin B	0,94	0,36	0,55	0,34	0,60
ApoB100	0,90	0,34	0,39	0,33	0,55
SERPING1	0,30	1,20	0,81	0,33	0,45
BLC / BCA-1 / CXCL13	0,60	0,76	1,01	0,33	0,78
SMPD4	1,89	0,38	0,37	0,33	0,26
ENPP2	0,42	0,63	0,61	0,33	0,35
AKAP9	3,15	0,24	0,66	0,33	0,68
Ribonuclease A	0,38	0,51	0,52	0,33	0,31
EN2	0,71	0,35	0,53	0,33	0,48
Glucagon	1,09	0,41	0,70	0,33	0,47
Desmoglein-1	0,59	0,58	0,65	0,33	0,78
MYO5A	0,46	1,26	0,58	0,33	0,19
DcR3 / TNFRSF6B	0,65	0,60	0,75	0,33	0,65

**LRP 4**	0,51	0,31	0,27	0,33	0,22
**Kremen-2**	0,78	0,52	0,54	0,33	0,50
**Gastrin**	1,08	0,36	0,58	0,33	0,23
**FABP3**	0,97	1,09	0,60	0,33	0,45
**TNF-alpha**	0,72	0,71	0,36	0,32	0,30
**EMAP-II**	0,36	0,50	0,55	0,32	0,45
**TPM1**	0,25	2,29	0,34	0,32	0,22
**Properdin**	0,88	0,28	0,31	0,32	0,16
**TNK1**	0,46	0,76	0,43	0,32	0,17
**CPN2**	0,49	0,26	0,80	0,32	0,43
**MN1**	0,19	1,34	0,60	0,32	0,35
**GCSH**	0,33	0,45	0,38	0,32	0,22
**FKBP12**	0,33	0,63	0,93	0,32	0,81
**DCXR**	0,44	0,44	0,55	0,32	0,31
**RNASE4**	0,20	0,46	0,52	0,32	0,18
**CLPS**	0,72	0,39	0,57	0,32	0,38
**CTLA-4 /CD152**	0,50	0,37	0,62	0,32	0,94
**HRG-beta 1**	0,18	0,42	0,21	0,32	0,31
**CCDC25**	0,54	1,08	0,96	0,32	1,04
**EDA-A2**	0,84	0,56	0,55	0,32	0,35
**GDI2**	2,45	0,25	0,39	0,31	0,48
**ITIH2**	0,41	0,82	0,46	0,31	0,57
**Itk**	0,39	0,29	0,65	0,31	0,20
**Desmocollin 1**	0,40	0,28	0,35	0,31	0,15
**ApoC1**	0,59	0,43	0,63	0,31	0,41
**Transaldolase 1/TALDO1**	0,26	0,65	0,41	0,31	0,30
**Dopamine beta Hydroxylase/DBH**	0,40	0,43	0,36	0,31	0,23
**Cytokeratin 18**	0,40	1,45	0,51	0,31	0,40
**Neuropilin-2**	0,99	0,61	0,97	0,31	0,68
**NME3**	1,74	0,97	0,36	0,31	0,31
**GITR Ligand / TNFSF18**	0,50	0,45	0,54	0,31	0,29
**Lubricin**	0,62	0,23	0,38	0,31	0,37
**Eps 15**	2,58	0,31	0,34	0,31	0,48
**LIMS1**	0,70	0,08	0,48	0,31	2,24
**Cryptic**	0,78	0,55	0,82	0,31	0,51
**HCFC1**	0,92	0,44	0,64	0,31	1,24
**Visfatin**	0,18	1,94	0,32	0,31	0,38
**Factor IX**	0,21	1,12	0,60	0,30	0,52
**GDF11**	0,68	0,47	0,49	0,30	0,56
**CCR3**	0,38	0,91	0,51	0,30	0,61
**Insulin**	1,44	0,99	0,30	0,30	0,32
**Collagen XV alpha 1**	0,38	1,43	0,55	0,30	0,77
**IL-2**	0,68	0,43	0,84	0,30	0,81
**Chromogranin C**	0,70	0,33	0,33	0,30	0,26
**ICAM-3 (CD50)**	1,52	1,57	0,00	0,30	0,89
**Dermcidin**	0,60	0,60	0,67	0,30	0,51
**LEKTI/SPINK5**	0,33	1,26	0,60	0,30	0,28

**Hck**	0,53	0,39	0,56	0,30	0,57
**Lyn**	0,34	1,03	0,64	0,30	0,48
**IL-7**	0,44	0,41	0,85	0,30	0,51
**SDPR**	0,96	0,48	0,60	0,30	0,41
**CD27 / TNFRSF7**	0,58	0,75	1,23	0,30	0,45
**GDF9**	0,60	0,59	0,72	0,30	0,60
**TCN1**	0,17	0,52	0,56	0,30	0,21
**PEBP4**	0,54	0,39	0,50	0,30	0,54
**CLC**	0,41	0,40	0,44	0,30	0,51
**Granzyme M**	0,35	0,83	0,60	0,30	0,42
**Endothelin**	0,74	0,65	0,70	0,29	0,82
**Proteasome 20S a+b**	1,16	0,42	0,50	0,29	0,25
**APM2**	0,55	0,16	0,51	0,29	0,49
**ARFBP1**	0,34	0,92	0,65	0,29	0,55
**PSMD9**	0,60	0,50	0,71	0,29	0,46
**CD30 / TNFRSF8**	0,77	0,61	0,79	0,29	0,53
**COL4A3**	0,94	0,39	0,00	0,29	0,00
**ITIH1**	0,43	0,65	0,63	0,29	0,48
**LAMP2**	0,27	0,21	0,25	0,29	0,24
**FGF-10 / KGF-2**	0,51	0,39	0,77	0,29	0,45
**Ntn1**	0,22	0,52	0,46	0,29	0,18
**Factor XII**	0,59	0,43	0,52	0,29	0,25
**IL-5**	0,49	0,62	0,83	0,29	0,71
**CFHR 1**	0,67	0,56	1,11	0,29	1,03
**EFTUD2**	0,34	0,47	0,46	0,29	0,26
**Glut2**	0,62	0,41	0,93	0,29	0,80
**IL-21**	0,50	0,83	0,61	0,29	0,80
**SOX4**	0,47	1,02	0,44	0,29	0,54
**BMX**	0,36	0,21	0,56	0,29	0,29
**EFEMP2**	0,42	0,66	0,71	0,28	0,22
**BTC**	0,65	0,58	0,56	0,28	0,33
**C9orf40**	0,41	0,68	0,75	0,28	0,44
**DRIL1**	0,39	0,38	0,28	0,28	0,21
**UFM 1**	0,21	0,84	0,45	0,28	0,22
**WISP2**	0,83	0,64	0,38	0,28	0,55
**ADH1C**	0,35	0,43	0,72	0,28	0,47
**gamma Catenin**	0,36	0,57	0,52	0,28	0,21
**Cytokeratin 10**	0,85	0,31	0,30	0,28	0,36
**HP1BP3**	3,12	0,39	0,21	0,28	0,51
**PTPRD**	0,25	1,33	0,52	0,28	0,86
**CV-2 / Crossveinless-2**	0,42	0,50	0,26	0,28	0,62
**FGF R5**	0,66	0,34	0,43	0,28	0,41
**Lysozyme**	1,50	0,57	0,49	0,28	0,24
**BIRC6**	0,21	0,81	0,52	0,28	0,44
**KRTDAP**	0,62	0,26	0,40	0,27	0,25
**Cytokeratin 17**	1,10	0,45	0,34	0,27	0,59
**Collagen III**	0,45	0,25	0,34	0,27	0,22

CPEB3	0,26	0,63	0,48	0,27	0,46
FER	0,37	0,23	0,52	0,27	0,20
HRG	0,59	0,68	0,25	0,27	0,30
53BP1	0,37	2,58	0,40	0,27	1,05
CPE	1,03	0,41	1,38	0,27	1,05
EphA5	0,20	1,74	0,63	0,27	0,63
Bax	0,89	0,47	0,47	0,27	0,96
SRMS	0,23	1,24	0,60	0,27	0,38
Aldolase C	0,40	0,60	0,64	0,27	0,26
GDF5	0,54	0,37	0,67	0,27	0,34
APCS	0,63	0,56	0,48	0,27	0,22
HPD	0,88	0,42	0,34	0,27	0,42
ASK1	0,37	0,24	0,46	0,27	0,20
IL-8	0,61	0,31	0,41	0,27	0,28
Apex1	0,20	1,23	0,39	0,27	0,18
CLIP170-N-t	0,51	0,47	0,66	0,27	0,35
Glutamyl hydrolase gamma /CGH	0,71	0,49	0,91	0,27	0,83
ALP	0,79	0,14	0,55	0,26	0,84
Eosinophil derived neurotoxin/EDN	0,63	0,42	0,79	0,26	0,35
TFF1	0,26	1,39	0,44	0,26	0,19
Rbm15	0,28	0,57	0,40	0,26	0,31
ITIH3	0,59	0,46	0,46	0,26	0,46
FDPS	0,98	0,31	0,22	0,26	0,37
Integrin b1	0,25	0,40	0,46	0,26	0,64
Envoplakin	0,52	0,28	0,52	0,26	0,24
PGLS-C-t	0,17	0,39	0,35	0,26	0,20
Ephrin B2	0,50	0,15	0,09	0,26	0,21
Thyroglobulin	0,23	0,93	0,70	0,26	0,19
IL-13	0,57	0,40	0,53	0,26	0,58
SPEN	0,15	0,49	0,32	0,26	0,26
VWF	0,16	0,48	0,72	0,26	0,31
IL-15	0,83	0,44	0,65	0,26	0,39
GDF3	0,51	0,39	0,60	0,26	0,48
Tyk2	0,26	2,24	0,43	0,26	0,38
Laminin b2	0,25	0,34	0,52	0,26	0,37
IFN-gamma	0,58	0,46	0,56	0,26	0,62
sFRP-3	0,28	1,17	0,33	0,25	0,63
HEXB	0,27	0,74	0,51	0,25	0,12
ADH1B	0,47	0,36	0,39	0,25	0,64
FGF-16	0,44	0,45	0,32	0,25	0,56
HSC70	0,86	0,33	1,21	0,25	3,21
BNP	0,72	0,86	0,29	0,25	0,12
Dkk-4	0,38	0,37	0,66	0,25	0,58
Ahsp	0,59	0,35	0,59	0,25	0,39
A1BG	0,48	0,30	0,59	0,25	0,32
FGF-20	1,11	0,50	0,59	0,25	0,47
Factor V	0,40	0,48	0,33	0,25	0,24

**MMRN1**	0,24	0,32	0,54	0,25	0,49
**ILK**	0,70	0,48	0,43	0,25	0,66
**GAPDH**	0,51	0,36	0,80	0,25	0,80
**ABCF1**	0,79	0,50	0,75	0,25	0,73
**Neogenin**	0,12	0,64	0,41	0,25	0,29
**P20Sb3**	0,12	0,64	0,45	0,25	0,12
**EphA8**	0,23	1,44	0,59	0,25	0,53
**IL-10**	0,53	0,29	0,59	0,24	0,67
**DAN**	0,75	0,51	1,06	0,24	0,62
**Tec**	0,20	1,57	0,42	0,24	0,47
**Frizzled-7**	0,31	1,07	0,34	0,24	0,59
**FRK**	0,66	0,80	0,56	0,24	0,33
**NELL2**	0,29	2,00	0,50	0,24	0,21
**IL-1 alpha**	0,61	0,48	0,60	0,24	0,57
**Calpain 1**	0,34	0,41	0,62	0,24	0,15
**Collagen X**	0,42	0,36	0,34	0,24	0,28
**CD44**	0,41	0,35	0,45	0,24	0,25
**PI 3Kinase p85 beta**	0,31	1,01	0,54	0,24	0,25
**ANP**	0,34	0,29	0,32	0,24	0,47
**hGH**	0,31	0,53	0,47	0,24	0,51
**Hemoglobin subunit gamma 2/HBG2**	0,54	0,30	0,44	0,24	0,48
**ADAMTS-19**	0,27	0,38	0,55	0,24	0,20
**CO4A2**	1,08	0,29	0,63	0,24	1,13
**AFG3L2**	0,47	0,23	0,65	0,23	0,32
**Inhibin beta**	0,27	0,31	0,46	0,23	0,24
**ABAT**	0,64	0,16	0,89	0,23	0,48
**ANK**	0,15	1,43	0,31	0,23	0,78
**NAIP**	0,29	0,74	0,50	0,23	0,30
**C8B**	0,52	0,35	0,38	0,23	0,21
**Ubiquitin**	0,18	0,44	0,26	0,23	0,34
**IBP160**	0,37	0,57	0,42	0,23	0,27
**SMAC**	0,21	0,85	0,59	0,23	0,14
**GPLD1**	0,18	0,67	0,42	0,23	0,28
**Insulin R**	0,86	0,82	0,41	0,23	0,44
**KIF3B**	0,90	0,56	0,45	0,23	0,25
**Omentin**	0,21	1,29	0,37	0,23	0,21
**FBP 38**	0,24	0,47	0,37	0,23	0,32
**Hornerin**	0,91	0,12	0,25	0,23	0,26
**URP2**	0,60	0,71	0,40	0,23	0,24
**PHAP1**	0,32	0,25	0,43	0,23	0,21
**LYVE-1**	0,22	0,22	0,36	0,23	0,99
**Fibrinogen gamma chain/FGG**	0,62	0,37	0,42	0,23	0,81
**SerpinB4**	0,16	1,21	0,48	0,23	0,25
**BAD**	0,18	0,62	0,48	0,23	0,29
**MMP-20**	0,28	1,16	0,35	0,22	0,47
**GARNL1**	0,28	0,44	0,40	0,22	0,15
**LZTS1**	0,16	1,46	0,42	0,22	0,19

**LTF**	0,26	0,45	0,46	0,22	0,33
**TYRO10**	0,30	1,17	0,62	0,22	0,34
**MSHa**	0,41	1,11	0,48	0,22	0,23
**MMP-19**	0,22	0,89	0,31	0,22	0,51
**Destrin**	0,38	0,20	0,67	0,22	0,16
**IRS2**	0,39	0,34	0,47	0,22	0,11
**Bcl-w**	0,26	0,82	0,27	0,22	0,36
**HSPA1A**	0,23	0,26	0,49	0,22	0,30
**Prostaglandin D Synthase/PTGDS**	0,69	0,24	0,35	0,22	0,15
**KCTD10**	0,22	1,40	0,37	0,22	0,30
**GREMLIN**	0,59	0,37	0,63	0,22	0,70
**Histone H1.2**	0,55	0,55	0,89	0,22	0,82
**CEP57**	0,26	0,64	0,46	0,22	0,07
**VGF**	0,26	1,04	0,66	0,22	0,11
**HMGB2**	0,09	0,59	0,60	0,21	0,30
**IL-6**	0,72	0,31	0,41	0,21	0,31
**CD 79 alpha**	0,24	1,64	0,44	0,21	0,20
**PYK2**	0,21	1,56	0,35	0,21	0,24
**CSH1**	0,31	1,25	0,45	0,21	0,22
**VDUP-1**	0,26	0,09	0,37	0,21	0,21
**BNIP2**	0,22	0,99	0,57	0,21	0,15
**Gelsolin**	0,33	0,56	0,37	0,21	0,21
**SET**	0,11	0,58	0,36	0,21	0,15
**EphB2**	0,18	1,20	0,57	0,21	0,26
**MDH1**	1,24	0,09	0,36	0,21	0,07
**GPCR GPR116**	0,32	0,40	0,49	0,21	0,22
**Aldolase B**	0,85	0,50	0,42	0,21	0,18
**Beta Defensin 4**	0,15	1,44	0,24	0,21	0,15
**FOXN3**	0,18	0,73	0,51	0,21	0,13
**EphA4**	0,22	0,42	0,43	0,21	0,15
**BCOR**	0,83	0,24	1,07	0,21	1,40
**Aminoacylase**	0,80	1,24	1,67	0,21	1,28
**GCSF**	0,38	0,32	0,52	0,21	0,38
**Oxytocin-neurophysin 1/OXT**	0,26	0,21	0,44	0,21	0,21
**MIG**	1,14	0,30	0,46	0,21	0,33
**RYK**	0,16	1,61	0,46	0,20	0,53
**CNDP1**	0,33	0,99	0,37	0,20	0,24
**POMC**	0,14	1,24	0,49	0,20	0,19
**IL-9**	0,48	0,30	0,52	0,20	0,40
**TRKB**	0,11	2,09	0,52	0,20	0,20
**Pancreastatin**	0,16	1,30	0,31	0,20	0,18
**EMILIN1**	0,49	0,61	0,45	0,20	0,26
**PGDF/PHGDH**	0,21	0,44	0,62	0,20	0,25
**Histone H2A.Z**	0,25	0,72	0,60	0,20	0,67
**HOXA10**	0,35	1,41	0,36	0,20	0,19
**DMRN9**	0,18	0,59	0,49	0,20	0,29
**LCAT**	0,62	0,24	0,29	0,20	0,10

**GM-CSF**	0,37	0,35	0,45	0,20	0,44
**CDK2**	0,24	0,39	0,58	0,20	0,32
**RELM alpha**	0,16	1,51	0,34	0,20	0,18
**FAK**	0,46	0,18	0,50	0,20	0,08
**GFR alpha-1**	0,41	0,62	0,46	0,20	0,95
**GHRF**	0,27	0,34	0,46	0,20	0,39
**EMSY**	0,72	0,84	0,62	0,20	0,99
**hnRNP A1**	0,30	1,57	0,82	0,20	0,38
**FAM20C**	0,36	0,26	0,59	0,20	0,92
**BD-1**	0,22	0,30	0,53	0,20	0,28
**DEFA6**	0,32	0,33	0,29	0,20	0,33
**Neuropeptide Y**	0,17	1,46	0,31	0,20	0,16
**pro-Glucagon**	0,21	1,83	0,30	0,20	0,27
**FKBP25**	0,17	0,23	0,86	0,20	1,34
**DMGDH**	0,38	0,22	0,48	0,19	0,25
**IL-17**	0,54	0,17	0,43	0,19	0,28
**beta B1 Crystallin/CRYBB1**	0,37	0,38	0,48	0,19	0,71
**Factor XIII B**	0,34	0,25	0,31	0,19	0,10
**ACLY**	0,32	0,30	0,77	0,19	0,32
**Alpha Lactalbumin**	0,22	0,25	0,47	0,19	0,31
**p23**	0,16	0,48	0,33	0,19	0,18
**MCP-1**	0,78	0,51	0,34	0,19	0,23
**Ankrd26**	0,18	0,62	0,28	0,19	0,35
**EphB3**	0,17	1,17	0,44	0,19	0,56
**MYHC**	0,30	0,36	0,57	0,19	0,32
**Nebulin**	0,47	0,50	0,36	0,19	0,18
**UGGT**	0,26	0,38	0,43	0,19	0,48
**Alanine Transaminase/ALT**	0,72	0,81	0,93	0,19	1,22
**Calretinin**	0,19	0,66	0,36	0,19	0,27
**Filamin A**	0,70	0,14	0,20	0,19	0,34
**BCHE**	0,13	1,10	0,26	0,19	0,74
**Nesfatin**	0,15	1,11	0,31	0,19	0,17
**CD 163**	0,81	0,27	0,31	0,19	0,21
**EPPK1**	1,49	0,22	0,42	0,19	0,48
**IGF2BP1**	0,44	0,81	0,37	0,19	0,13
**LOX**	0,45	0,26	0,36	0,19	0,08
**Ghrelin**	0,16	0,95	0,30	0,18	0,18
**Argininosuccinate Lyase/ASL**	0,31	0,06	0,28	0,18	0,35
**hnRNP L**	0,48	1,25	0,50	0,18	0,56
**KIAA1468**	0,31	0,16	0,43	0,18	0,16
**BTD**	0,26	0,70	0,45	0,18	0,22
**IGF2BP2**	0,16	0,60	0,58	0,18	0,23
**FIH**	0,53	0,92	0,36	0,18	0,13
**CCK**	0,15	1,71	0,27	0,18	0,21
**PRG2**	0,25	0,56	0,38	0,18	0,23
**KIAA1967**	0,38	0,51	0,41	0,18	0,19
**GDF8**	0,66	0,35	0,87	0,17	0,36

**AIF**	0,24	1,52	0,28	0,17	0,57
**PACS1**	0,12	0,53	0,58	0,17	0,23
**KRT85 - N-terminal**	0,20	0,44	0,36	0,17	0,28
**LAMP1**	0,30	0,50	0,51	0,17	1,30
**Apelin**	0,13	0,76	0,22	0,17	0,14
**Musk**	0,29	0,36	0,40	0,17	0,10
**PRDM13**	0,16	0,19	0,35	0,17	0,26
**LCMT2**	0,19	0,85	0,32	0,17	0,22
**LTK**	0,15	1,13	0,47	0,17	0,23
**ApoB**	0,31	0,28	0,31	0,17	0,44
**BRSK1**	0,45	0,46	0,56	0,17	0,34
**BID**	0,20	0,79	0,34	0,17	0,26
**PGRPL**	0,19	0,12	0,32	0,16	0,16
**LDHB**	0,15	0,46	0,37	0,16	0,25
**Corticosteroid-binding globulin**	0,37	0,53	0,48	0,16	0,46
**NRG3**	0,19	0,26	0,61	0,16	0,16
**Fibrinogen-like 2**	1,21	0,32	0,84	0,16	1,01
**C7**	0,23	0,67	0,43	0,16	0,16
**Androgen Receptor**	0,17	0,72	0,37	0,16	0,26
**Collagen IVa6**	1,34	0,33	2,16	0,16	0,49
**Complement Factor B**	0,84	0,85	0,52	0,16	1,31
**KRTHA3B**	0,27	0,10	0,21	0,16	0,22
**ETL**	0,28	0,30	0,59	0,15	0,16
**EphA7**	0,33	0,19	0,43	0,15	0,13
**MBL-2**	0,24	0,21	0,45	0,15	0,21
**PTMA**	0,16	0,25	0,40	0,15	0,17
**Glycerol 3 Phosphate Dehydrogenase**	0,26	0,20	0,39	0,15	0,21
**NPTX1**	0,11	1,25	0,43	0,15	0,22
**ASPH**	0,22	0,25	0,39	0,15	0,15
**Lck**	0,44	0,20	0,35	0,15	0,21
**MSH6**	0,19	0,28	0,43	0,15	0,20
**MIP 2**	0,24	0,34	0,41	0,15	0,19
**Endostatin**	0,10	0,52	0,35	0,15	0,11
**PLS3**	0,19	0,20	0,43	0,15	0,17
**Plxdc2**	0,26	0,06	0,31	0,15	0,11
**ASGR2**	0,47	0,38	0,51	0,14	0,27
**CD41**	1,14	1,05	1,57	0,14	2,10
**FOLR3**	1,12	0,76	0,61	0,14	1,15
**Vasopressin**	0,11	1,88	0,29	0,14	0,15
**EXTL2**	0,27	0,88	0,41	0,14	0,23
**NPAS3**	0,12	0,14	0,24	0,14	0,14
**ZBTB4**	0,17	0,20	0,30	0,14	0,19
**C9**	0,23	0,29	0,37	0,14	0,18
**HADHA**	0,23	0,42	0,37	0,14	0,11
**CFL1**	0,55	0,65	0,84	0,13	0,43
**Hepcidin**	0,11	0,86	0,22	0,13	0,14
**Neuropeptide B**	0,21	0,21	0,45	0,13	0,23

**IRE1**	0,16	0,25	0,38	0,13	0,19
**TGF-beta RI / ALK-5**	0,00	1,77	0,11	0,13	0,91
**Actinin alpha 1**	0,78	0,43	0,54	0,13	1,80
**EphB1**	0,40	0,50	0,36	0,13	0,12
**LOXL1**	0,23	0,24	0,34	0,13	0,20
**COL9A3**	0,30	0,75	0,66	0,13	0,18
**PYY**	0,11	1,01	0,25	0,12	0,14
**ESR1**	0,26	0,25	0,31	0,12	0,28
**Cadherin 22**	0,81	0,13	0,63	0,11	0,57
**CD32**	0,14	0,19	0,28	0,11	3,25
**CRTH-2**	0,62	0,16	0,41	0,11	0,27
**FCGBP**	0,73	0,36	0,39	0,11	0,77
**LTBP4**	0,17	0,28	0,44	0,11	0,08
**DCI**	0,67	0,12	0,48	0,11	0,28
**IGFBP7**	0,16	0,31	0,22	0,11	0,20
**NRG2**	0,14	0,24	0,34	0,10	0,13
**CNTF R alpha**	0,44	0,48	0,28	0,10	0,59
**CKB**	0,36	0,19	0,57	0,09	0,29
**MMP-24 / MT5-MMP**	0,06	0,15	0,22	0,08	0,31
**EPB41**	0,67	0,12	0,22	0,08	0,27
**AKR1B1**	0,10	0,20	0,22	0,07	0,22
**GBE1**	0,90	3,07	0,64	0,07	0,52
**DPP3**	0,55	0,34	0,48	0,07	0,47
**Laminin gamma 1**	0,39	0,04	0,03	0,07	0,01
**NABC1**	0,06	0,04	0,09	0,06	0,09
**COLEC10**	0,72	0,58	1,13	0,04	0,37
**CYTL1**	0,44	0,24	0,58	0,04	0,22
**ACTBL2**	0,46	0,05	0,56	0,02	0,21
**hnRNP A2B1**	0,23	1,24	0,08	0,00	0,25
**Tryptophanyl**	0,58	0,04	0,86	0,00	0,36
**Frizzled-1**	0,73	0,55	0,15	0,00	0,70

Supplementary data 1:

Results of the proteomic array of the pool groups, represented as fold changes. The results are sorted as far as possible from highest to lowest fold change of the mean values.

Since there are multiple values for each protein due to the analysis of different tumor stages, it is difficult to sort the values in a convenient way.

Proteins that were further validated by our group are highlighted in green.
